# The hidden costs of dietary restriction: Implications for its evolutionary and mechanistic origins

**DOI:** 10.1126/sciadv.aay3047

**Published:** 2020-02-21

**Authors:** Andrew W. McCracken, Gracie Adams, Laura Hartshorne, Marc Tatar, Mirre J. P. Simons

**Affiliations:** 1 Department of Animal and Plant Sciences and Bateson Centre, The University of Sheffield, Sheffield S10 2TN, UK.; 2 Department of Ecology and Evolutionary Biology, Brown University, Providence, RI, USA.

## Abstract

Dietary restriction (DR) extends life span across taxa. Despite considerable research, universal mechanisms of DR have not been identified, limiting its translational potential. Guided by the conviction that DR evolved as an adaptive, pro-longevity physiological response to food scarcity, biomedical science has interpreted DR as an activator of pro-longevity molecular pathways. Current evolutionary theory predicts that organisms invest in their soma during DR, and thus when resource availability improves, should outcompete rich-fed controls in survival and/or reproduction. Testing this prediction in *Drosophila melanogaster* (*N* > 66,000 across 11 genotypes), our experiments revealed substantial, unexpected mortality costs when flies returned to a rich diet following DR. The physiological effects of DR should therefore not be interpreted as intrinsically pro-longevity, acting via somatic maintenance. We suggest DR could alternatively be considered an escape from costs incurred under nutrient-rich conditions, in addition to costs associated with DR.

## Teaser

Dietary restriction leads to unexpected mortality increases when nutrient conditions improve, questioning evolutionary theory.

## INTRODUCTION

Ageing has attracted extensive scientific interest, from both a fundamental and biomedical perspective. Dietary restriction (DR) extends health and life span across taxa, from baker’s yeast to mice, with very few exceptions (*1*, *2*). The reduction in total calories—or restriction of macronutrients, such as protein—extends life span reliably (*3*–*5*). Although the precise universal mechanisms that connect DR to ageing remain elusive, translation of DR’s health benefits to human medicine is deemed possible. The widespread assumption of DR’s translational potential originates from the notion that DR’s beneficial effects are facilitated by shared evolutionary conserved mechanisms, as beneficial effects of DR are observed across taxa. Experiments on our close evolutionary relatives, rhesus monkeys (*Macaca mulatta*), have demonstrated that DR could be translational (*6*). Still, the mechanisms by which these benefits are accrued physiologically may differ between species, as no single genetic or pharmaceutical manipulation mimicking the benefits of DR across model organisms exists (*7*). In addition, genetic heterogeneity within species presents an additional layer of complexity, since efficacy of DR-driven longevity extension can differ between genotypes (*8*, *9*). Mechanistic insight will be key, since DR as a human lifestyle intervention has limited scope, given the degree of self-restraint required. It is therefore warranted to direct scrutiny toward the evolutionary theory of DR, since it underpins the assumed universality of physiological mechanisms by which DR confers health benefits.

Shared universal mechanisms can only be inferred from the ubiquity of the DR longevity response in the animal kingdom, when the selection pressures responsible for such evolutionary conservation are understood. The DR response itself may have evolved once, and mechanisms might be conserved. Alternatively, DR could have undergone convergent evolution, either using similar mechanisms—or by adopting alternative ones (*10*). These evolutionary scenarios provide distinct predictions as to how informative mechanistic research in other animals will prove for human medicine. Only if the DR response is rooted in ancient physiology (i.e., evolved once or through convergent evolution) can possible translation of mechanistic research on model organisms be confidently inferred. The DR effect itself is interpreted as an evolved, adaptive, pro-longevity physiological response to limiting food availability (*11*). Life history theory (*12*)—a central tenet of evolutionary biology—states that resources are limited, and thus predicts trade-offs between reproduction and survival, even in nutrient-rich environments. As such, DR presents an enigma: Why do organisms live longer on a constrained energy budget?

**Fig. 1:**
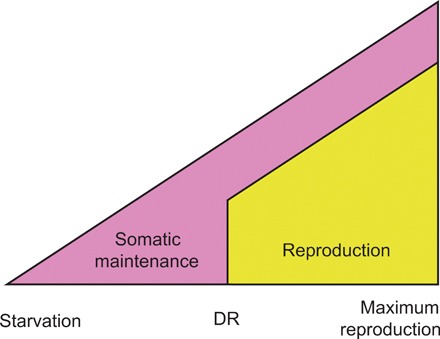
Schematic of the evolutionary model of DR. Resource availability is varied from left to right, from very low (where starvation would occur) to very high (where maximum reproduction would occur). The theoretical optimal allocation to somatic maintenance (pink) versus reproduction (yellow) is depicted at a given resource availability. When resource availability decreases, investment in both somatic maintenance and reproduction is reduced until a threshold is met. Below this point, resources are so scarce that investment in reproduction does not yield a fitness return. This could occur when offspring produced cannot recruit into the population due to the harsh resource environment, or because the capital (start-up) costs of breeding cannot be met. Here, investment in reproduction is lost and is wholly allocated to somatic maintenance. It is this evolved resource allocation decision to invest into somatic maintenance under DR conditions that is thought to underlie life-span extension under DR.

The currently accepted evolutionary model for DR (*13*, *14*) uses a life-history perspective on ageing to explain this enigma. The model proposes that below a certain resource threshold, organisms will reallocate energy almost exclusively toward somatic maintenance (Fig. 1). In certain ecological situations (e.g., severely reduced juvenile survival, or when the energy budget is lower than the initial costs, or the cost of one unit of reproduction), investment into reproduction will cease to yield fitness. The optimal, fitness-maximizing strategy under these harsh conditions would be to terminate investment into reproduction and use this energy to gain fitness when conditions improve. Crucially, this life history strategy would favor an increase in resources devoted to maintenance and repair during DR—allowing organisms to survive bouts of famine with an intact or superior soma (*13*, *14*). This “somatic maintenance response” has been presumed to be the primary causative agent in the pro-longevity DR response (*1*, *15*, *16*). There are few alternatives to the somatic maintenance response model that can explain the evolutionary biology of DR [but see (*17*–*19*)], and its elemental phenotypic predictions have undergone minimal empirical examination [but see (*20*)].

This attractive evolutionary rationale has given credibility to the assumption that physiological changes in the DR animal are inherently pro-longevity, since it implies DR increases investment into somatic maintenance. For example, transcriptomic up-regulation of what could be interpreted as maintenance and repair processes under DR has lent credence to this hypothesis (*15*, *21*, *22*). Directionality of these associations is often ambiguous, however, as, for example, down-regulation of DNA repair under DR could be interpreted as either a reduction in DNA damage generation or reduced investment into repair (*21*, *22*). In other words, a potentially simpler rationale is often neglected: the surge of “maintenance and repair” gene expression as a mere stress response to metabolic disruption. The health benefits observed under DR might originate from a passive response—one not necessarily evolved as an adaptive regulatory response that increases somatic maintenance in response to DR. Under these circumstances, life-span extension could be a simple correlated response to currently unknown, but strongly conserved, physiology. For example, the limitation of metabolic rate or reduction in specific metabolites as a direct consequence of DR could reduce conserved associated physiological dysfunction and thereby extend life span. The negative physiological effects suffered by dietary- restricted organisms, e.g., compromised immune function (*23*) and cold intolerance (*17*), could arise from a similar passive response and are not necessarily the result of a regulated trade-off. DR is sometimes considered a hormetic response—mild stress, resulting in the stimulation of conserved cellular reactions leading to beneficial health (*24*)—which would be a similar example of a passive response. One example of such a hormetic response is the activation of heat shock proteins, which show only very transient expression but long-lasting effects on life expectancy (*25*).

The distinction between passive-correlated versus adaptive-programmed pro-longevity responses will be key to identifying the mechanisms of DR and develop translation to humans. The current, widely accepted evolutionary model of DR (*13*, *14*) supports an adaptive phenotypic response and provides a key prediction: Organisms should increase investment into their soma during periods of DR, and therefore, when their resource availability improves, should outcompete age-matched rich-fed controls in survival and/or reproduction. Here, we provide an experimental phenotypic test of this prediction using a large-scale demographic approach detailing mortality and fecundity in *Drosophila melanogaster* fed different dietary regimes. Our results revealed substantial mortality and fecundity costs when returning to a rich diet after a period of DR, falsifying the key prediction provided by the evolutionary biology of DR. These effects were independent of genotype, duration of DR, and number of dietary fluctuations, and we excluded large confounding effects arising from access to water (*26*), the social environment (*27*), the microbiome (*28*), and sex (*29*). Our results therefore suggest that the effects of DR are not necessarily intrinsically pro-longevity, i.e., by increasing investment into somatic maintenance, and could alternatively be considered an escape from costs incurred under nutrient-rich conditions and/or costs associated with DR. These insights question the relevance of the somatic maintenance explanation of DR in guiding biomedical research into its mechanisms. Our alternative paradigm—a passive, not necessarily directly adaptive response to DR—gives renewed credibility to a range of mechanistic hypotheses of DR: hormesis (*30*), a reduction in metabolism causing reduced oxidative damage generation (*10*, *31*) and improved mitochondrial functioning (*32*), or a reduction in waste products from specific metabolic pathways (*33*).

## RESULTS

### Hidden costs of DR

The use of large populations of animals, possible in the fruit fly and other small organisms, allows the measurement of age-dependent mortality risk—the risk to die at a given age. Such a demographic approach can be useful to infer underlying biology (*34*, *35*) and can be used experimentally to investigate instantaneous effects of treatments on mortality (*36*, *37*). We used an experimental demographic approach comprising 11,084 individual deaths (table S1) to test the phenotypic predictions from the evolutionary theory of DR: Increased investment in somatic maintenance under DR allows the animal to better perform when nutrient availability improves.

**Fig. 2:**
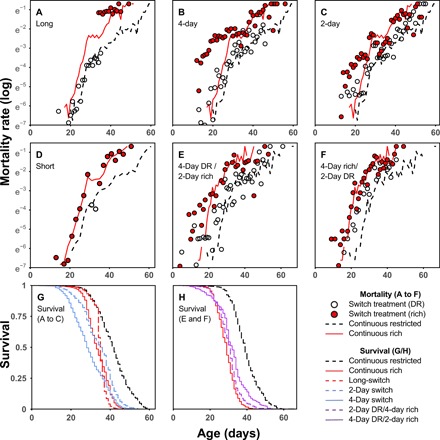
The effect of different dietary regimes on age-specific mortality risk in DGRP-195. Age-specific mortality risk (**A** to **F**) allows an investigation of instantaneous changes in mortality risk upon dietary switches (points) across the different dietary regimes used. Mortality risk at continuous rich (solid red) and restricted diets (dash black) are plotted as lines. The exacerbation of mortality due to switch phenotypes is the difference between mortality at continuous rich diet (red line) and mortality of switch treatment when on a rich diet (red points). The open dots in the switch treatment, the DR condition, should overlay the continuous DR treatment (dash) if the dietary switch does not modulate the effect of the DR diet [or act as a pure control before a single switch, as in (A)]. *N* = 19,086 females total; 995 to 3769 per treatment. (A) Long-switch. When returning to a rich diet after a long period of DR, mortality is exacerbated compared with flies fed a rich diet continuously. (B) Four-day switch. Switching from a DR to a rich diet repeatedly every 4 days increases mortality on rich diets compared with continuously rich-fed flies. Flies are still able to modulate their mortality in response to DR even when diet fluctuates rapidly. (C) Two-day switch. Mortality on rich diets is only mildly increased and flies still respond to DR even when it is only imposed for 2 days. (D) Short reverse-switch. After a long period on a rich diet, DR for 4 days returns flies to mortality of continuous DR. The *x* axis of (D) is age adjusted to correct for age differences (1 to 3 days) at the time of the diet switch for illustration purposes only. (E) Four-day DR, 2-day rich switch (4-to-2–day switch). Flies respond to DR but encounter a slightly blunted effect compared with continuous DR. (F) Four-day rich, 2-day DR switch (4-to-2–day switch). The effect of DR is reduced when imposed for 2 days following 4 days on a rich diet. (**G**) Survival plot of (A) to (C) with associated continuous diet controls. Total survival of both the 4-day switching dietary regime and the long-switch is lowered compared with continuously rich diets, despite flies spending a considerable extent of their lives on restricted diets. Flies on DR outlive all other categories. (**H**) Survival plot of (E)/(F) with associated continuous diet controls. Despite spending up to two-thirds of their lives on DR in these asymmetrical regimes, survival benefits are modest, compared with continuous DR. Dietary switch treatments contain daily time points (dots) for the dietary switch treatments, as treatments were mirrored and balanced, with half of flies starting on DR and half on rich diets.

DR imposed continuously throughout adult life resulted in a significant reduction in mortality rate (Fig. 2 and tables S1 and S2; *P* < 0.001, three times lower hazard). In addition, switching flies to DR at older ages instantly reduced mortality levels to the levels of flies that had experienced continuous DR (“short reverse-switch”; Fig. 2D and table S1). Such mortality amnesia—a complete absence of historic diet effects—has been reported previously in flies (*36*, *37*).

Our expectation, based on the current evolutionary model of DR, was that if flies were returned to rich food conditions after a period of DR, they would have a superior soma compared with flies that experienced rich food continuously. Resources allocated to somatic maintenance should result in higher fitness (Fig. 1). In contrast, our “long-switch” treatment resulted in a substantial increase in mortality risk compared with flies kept on a rich diet throughout life (Fig. 2A and table S1; *P* < 0.001, 3.7 times higher hazard). Mortality peaked immediately (within 48 hours; 5.1 times higher hazard) after the switch from a restricted to a rich diet. The magnitude of this mortality difference decreased slowly thereafter, resulting in no difference between the continuous rich diet and the long-switch treatments after 8 days (Fig. 2A and table S3; *P* < 0.001).

### Repeated diet switching

The long-switch dietary treatment could be dependent on several specific aspects of the imposed dietary regime, and this would not necessarily falsify the somatic maintenance hypothesis of DR. First, the effects of the long-switch treatment could be contingent upon the prior duration of DR. It has been suggested that DR evolved in response to relatively short, intermittent bouts of famine (*13*, *14*). Second, it has been suggested that the longevity response to DR originated from selection pressures on relatively young individuals (*13*). Thus, younger flies might not show the heightened mortality we observed. Third, it could be that sudden changes in diet per se are harmful. To test these three potential confounds, we used short recurring bouts of DR, alternating between a rich and a DR diet every 4 days (“4-day switch”). In this dietary regime, mortality on the rich diet compared with the continuous rich diet was similarly exacerbated (Fig. 2B and tables S1 and S2; *P* < 0.001, 2.4 times higher hazard). This 4-day switch dietary regime also allowed us to examine whether flies were able to instantly and repeatedly modulate their mortality risk in response to diet, similar to the short reverse-switch treatment (Fig. 2D). Flies indeed modulated their mortality in response to the diet they were currently fed, with a degree of unexpected immediacy. Mortality risk on DR, within the 4-day switch regime, repeatedly decreased to levels similar to that of flies continuously exposed to a restricted diet (Fig. 2Band table S1). Nonetheless, mortality risk during these periods of DR imposition was significantly higher than that of continuous DR-treated flies (table S1; *P* < 0.001, 1.6 times higher hazard). We suggest this increase in mortality seen on DR in the 4-day switch treatment is due to either accrued physiological costs or more probable, a carryover of deaths directly resulting from the rich diet, but recorded on the DR diet.

### Mortality costs depend on the duration of DR

A closer examination of the timing of mortality within the 4-day switching paradigm showed that the mortality response was strongest in the second 48 hours after exposure to both DR and rich diets (table S4; *P* < 0.001). This suggests a period of acclimation to both DR and rich diets is necessary before their physiological effects are fully realized. To test the importance of the duration of exposure to DR and rich diets for the mortality phenotypes observed, further dietary regimes were used. First, switching from DR to rich conditions was carried out at increased frequency—alternating every 2 days (“2-day switch”; tables S1 and S2). This 2-day switch dietary regime confirmed that sustained exposure to the diets (longer than 2 days) was required to cause the full magnitude of the mortality phenotypes observed. On a rich diet, the 2-day switch regime showed slightly higher mortality compared with the continuous rich diet (Fig. 2C; hazard = 1.1, *P* < 0.05), and mortality on DR in the 2-day switch regime did not reduce to the levels seen in continuously dietary-restricted flies (Fig. 2C; hazard = 1.3, *P* < 0.001). Together these diet-specific mortality effects resulted in an overall life-span extension in the 2-day switch regime (Fig. 2G and table S2; *P* < 0.001). As flies spend an equal amount of time on DR or rich diets in the 2-day switch regime, the reduction in mortality under DR can be considered to be relatively more rapid than the induction of exacerbated mortality on rich food (after a period of DR). We reasoned that the exacerbation of mortality on rich food requires an extended period on either restricted or rich food. To test this directly, asymmetrical dietary regimes were used.

In this additional set of experiments, we combined the 4- and 2-day switching regimes: Treatments were composed of 4 days on either a DR or rich diet, followed by 2 days on the other (“‘4-to-2–day switch”). Similar to the 4-day switch, this dietary regime was repeated sequentially. These “4-to-2” regimes showed no marked increase in mortality on the rich diet compared with flies on a continuous rich diet (Fig. 2, E and F, and table S5). Relative to a continuous DR treatment, the effect of DR within this paradigm was markedly reduced, especially when flies were restricted for 2 days only (Fig. 2F and table S5). This reduction in the mortality response to DR in the 4-to-2 regimes amounted to a marked reduction in the total longevity extension achieved when compared with continuous DR. When flies spend two-thirds of their lives on DR, life span was only extended by half (compared with continuous DR), and only a quarter when flies spend one-third of their lives on DR (Fig. 2, E and F, and table S6). These experiments again suggest a period exceeding 2 days on either diet is required to induce marked mortality effects.

Note that within the long-switch treatment, the mortality exacerbation observable on rich food was strongest within the first 2-day interval (Fig. 2A and table S3). In addition, our short reverse-switch induced a full DR response—mortality amnesia—within 2 days (Fig. 2D and table S1). Moreover, the ameliorated mortality exacerbation of our additional switch experiments (2- and 4-to-2–day switches) strongly suggests that the sudden dietary perturbations themselves are not the cause of premature mortality in our switching regimes. From these combined results, we therefore conclude that the additional mortality costs observable on a rich diet are contingent upon the prior duration of DR. The increase in mortality when resource availability is reinstated, we report here, is in direct contrast to DR having evolved as a life history strategy to invest into somatic maintenance to prepare for times when resources are plentiful again.

### Genetic variance

**Fig. 3:**
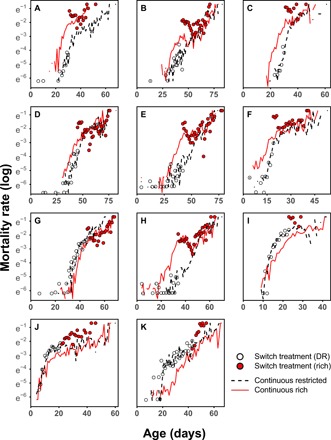
Long-switch treatment in a panel of 11 DGRP genotypes. (**A**) 195, (**B**) 105, (**C**) 217, (**D**) 441, (**E**) 705, (**F**) 707, (**G**) 136, (**H**) 362, (**I**) 239, (**J**) 335, and (**K**) 853. *N* = 29,702 females total; ~2725 females per genotype; 13,375 for continuous rich treatments, and ~8170 each for the two other treatments. The dietary switch for the long-switch treatment group occurred at 45 to 65% of continuous rich treatment flies. All panels contain daily time points as in Fig. 2. Exposure to a high-nutrient diet after a period of DR resulted in marked increase in mortality compared with a continuous rich diet in all lines (9 of 11 significant). There was genetic variation in this response, with DGRP-136 (G) and DGRP-362 (H) showing the smallest effects. This marked overshoot was not contingent upon DR extending life span. Lines that showed “starvation” on a DR diet still showed significant overshoots when they were switched to a rich diet, where recovery from starvation was expected, even when compared with continuous DR diets (I to K)

**Fig. 4:**
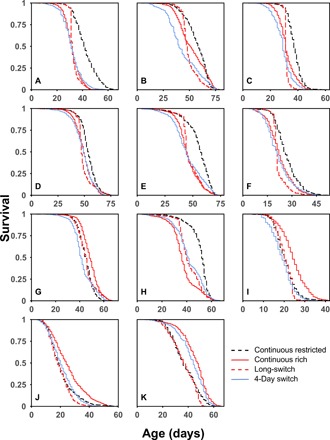
Survival curves of DGRP panel for both dietary regimes. (**A**) 195, (**B**) 105, (**C**) 217, (**D**) 441, (**E**) 705, (**F**) 707, (**G**) 136, (**H**) 362, (**I**) 239, (**J**) 335, and (**K**) 853. Total survival on the different dietary regimes across the genetic panel tested. Rich diets after a period of DR resulted in such an increase in mortality, that total survival of the cohort was lower (or equal to) than those fed a continuous rich diet for their whole life (A to F). *N* = 37,897 females total; ~3450 females per genotype; 13,375 for continuous rich treatments, and ~8170 for all other treatments.

The above set of diet experiments were conducted using the wild-type inbred lineage DGRP-195. To eliminate the possibility that the dietary responses described above were the result of rare genetic effects inherent to this specific genetic line, we performed the same dietary perturbations in a panel of randomly selected inbred genotypes (DGRP-105, 136, 195, 217, 239, 335, 362, 441, 705, 707, and 853). Across our panel, we detected an increase in longevity under DR conditions (Figs. 3 and 4; additive model, DR hazard = −0.21 ± 0.08, *P* < 0.001). There were considerable genetic effects in response to diet; however [interaction model: χ^2^ = 204.8 (*df* = 10), *P* < 0.001], with some genotypes showing elevated mortality under restricted-diet conditions, compared with continuously fed rich diet flies (Figs. 3 and 4). This degree of variation in response to DR can be explained by genetic variation in the reaction norm to diet and not necessarily as an absence of the longevity response to DR. Animals react to an increasing degree of food restriction by first reducing reproduction and then by increasing life span—the DR longevity response. Further food restriction, beyond the nutritional optimum for longevity, decreases life span through starvation. A particular combination of one restricted and one rich diet will therefore not always induce the same longevity response in a range of genotypes, when these genotypes differ in their reaction norm to diet (*8*, *9*).

Across genotypes, exposure to the rich diet after a period of DR (long-switch) resulted in exacerbated mortality, exceeding that of flies fed a rich diet for their whole lives (Fig. 3; additive model, hazard = 0.997 ± 0.056, *P* < 0.001). There was significant genetic variance for this trait [χ^2^ = 124 (*df* = 10), *P* < 0.001]. Still, all genotypes showed a mortality overshoot, compared with a continuous rich diet, following a switch from DR to high-nutrient conditions (Fig. 3 and tables S7 and S8; 9 of 11 significant; range, 1.12 to 5.21 times hazard). Genetic variation assessed in a larger amount of lines could be used to uncover the associated mechanisms, but our objective here was to exclude the possibility of rare genetic effects inherent to a single line being responsible for the phenotypes we observed. Alternating the diet from DR to rich every 4 days decreased longevity compared with the continuous rich diet, across the genetic panel (additive non–interval-based model, hazard = 0.24 ± 0.047, *P* < 0.001). Again, we found significant genetic variance for the response to this dietary regime [χ^2^ = 117 (*df* = 10), *P* < 0.001]. Lines differed in their responses: 5 of 11 showed marked decreases in survival, 1 showed an increase in survival, and the remaining 5 showed statistically nonsignificant effects (table S8). Interval-based models showed that mortality rates increased at the rich diets following a period of DR, as in the long-switch, in all lines (significant in 7 of 11; tables S10 and S11). There was a modest positive genetic correlation in the increase in mortality induced by the long-switch and 4-day switch dietary regimes (correlation of coefficients from tables S9 and S11; *r*_s_ = 0.45, *P* = 0.17), suggesting these dietary phenotypes originate from similar physiology.

### Hidden costs: Independent of a pro-longevity DR response

Our restricted diet unexpectedly induced a putative starvation response—observable as an increased mortality rate—in four lines (136, 239, 335, and 853; Figs. 3 and 4, and tables S7 and S9 to S11). These contrasting responses to DR serendipitously allowed us to see whether the dietary switching phenotypes were contingent on the direction of the DR response. Unexpectedly, when lines that showed starvation were refed on a rich diet (long-switch), mortality did not decrease but increased (tables S7 and S8; three of four showed a significant increase), even beyond the heightened mortality seen on DR (Fig. 3 and table S12). Similarly, within the 4-day switching regime, mortality risk was exacerbated at a rich diet. The pattern of mortality even reversed, compared with individuals fed diets continuously, with lines now showing a putative DR longevity response within the 4-day switch dietary regime (fig. S1 and tables S10, S11, and S13). These outcomes were particularly remarkable since exposure to a richer diet was expected to rescue the starvation response. In contrast to any recovery upon a return to a rich diet, individual mortality risk surged beyond that of flies fed rich diets continuously. These observations further fit with our interpretation that the dietary responses we report here are general in flies and are not contingent on the phenotypic pro-longevity response to DR. In addition, the reversal of the mortality patterns in the 4-day switch regime suggests differences in the reaction norm to nutrient restriction, as discussed above, could be largely responsible for the genetic variance in the DR longevity response we observe. We find that short bouts of refeeding on this dietary regime present genetic lines normally experiencing starvation on DR the opportunity to overcome malnourishment and extend life span. This indicates that these specific genetic lines are not refractory to the life-span extension effects of DR but are merely more susceptible to low-nutrient conditions. Given this, we predict that the starvation that these lines exhibit under DR would, under slightly higher-nutrient conditions, result in a pro-longevity DR response.

### Cost of mortality not compensated for by fecundity increase

We recognized that our results would not necessarily discredit the evolutionary model of DR should the observed costs in mortality be compensated fully, or partially, by an increase in fecundity. Egg production across the DGRP panel experiment was measured from vials in each dietary regime and expressed both as a total count (age-specific fitness of the population; figs. S2 and S3 and tables S14 and S16) or eggs per fly (age-specific reproductive output, corrected for mortality differences; figs. S2 and S3 and tables S15 and S17). All lines responded strongly to DR by reducing reproductive output. Within the 4-day switching paradigm, DR also induced a rapid reduction in fecundity (fig. S3 and tables S16 and S17). As with the mortality response, genetic lines also differed in fecundity response to the dietary treatments [long-switch: *F* = 57 (*df* = 2), *P* < 0.001; 4-day switch: χ^2^ = 187 (*df* = 9), *P* < 0.001]. However, in both metrics, our switching diets underperformed in reproductive output compared with the continuous rich diet (figs. S2 and S3 and tables S14 to S17), confirming our mortality phenotypes were not compensated by higher fecundity upon a return to nutrient-rich conditions.

### Mortality phenotypes were not contingent on condition of the microbiome, social housing, water, or sex

A switch to rich diets after a sustained period of DR (long-switch) still resulted in an increase of mortality when flies were treated with antibiotics (table S18; *P* < 0.001), provided additional water (table S19; *P* = 0.002), or when mortality was assessed in isolation (table S20; *P* = 0.014). Males responded, similarly to females, by increasing mortality on rich diets if this was preceded by 4 days of DR (4-day switch, table S21; *P* = 0.001, long-switch not tested).

## DISCUSSION

DR has been tested across multiple species, and the resulting life-span extension has consistently—with very few exceptions (*17*)—been interpreted as provoking anti-ageing, pro-longevity physiology. This interpretation is based on the widely accepted evolutionary theory of DR (*13*, *14*), which predicts that during periods of DR, investment in somatic maintenance is actively increased, to await better times when fitness can be gained. In contrast, we find that periods of DR did not result in a superior soma and instead resulted in large increases in mortality and reductions in fecundity, when nutrient availability returned to plentiful. Our results question the current explanation of DR’s evolutionary origins and, thereby, its relevance in interpreting DR’s mechanistic origins.

Other studies have raised similar concerns but have only very rarely measured the consequences of the relevant life history event: a period of DR followed by a period of rich-food conditions. Direct measurement of investment into the soma using stable isotopes showed no increased investment under DR (*38*). Experimental evolution across 50 generations under DR failed to support the current evolutionary theory of DR (*20*). Further lack of support, we suggest, originates from the remarkably immediate reduction in mortality—a reduction in frailty, rather than actuarial ageing rate (*34*, *36*, *37*) or historic physiological effects of diet—seen when flies are dietary restricted. A limited number of previous studies with *Drosophila* have shown such a response (*36*, *37*). We confirmed these results (Fig. 2D) but also show that flies are capable of reducing mortality repeatedly, in response to multiple switches in diet (fig. S1). Since DR does not slow ageing demographically but results in an instant lowering of mortality—without any accrued beneficial effects—this is in itself evidence against increased somatic investment under DR (*34*).

In the reverse scenario, when flies resumed rich diets after DR, their performance was markedly lower than that of flies that were fed rich diets for their entire lives. Notably, this effect held even when DR caused starvation—resulting in exacerbated mortality on the diet that should have provided an opportunity to refeed. Previous studies did not detect the same mortality costs in dietary regimes analogous to our long-switch (*36*), although in the raw nonsmoothed data, some exacerbation of mortality can be seen in some conditions. There are a number of potential variables, which could explain these differences. First, the duration of DR before a rich diet appears to be integral to inducing exacerbated mortality on rich diets (Fig. 2). Second, the existence and intensity of both the long-switch and 4-day switch phenotype are genotype dependent (Fig. 3and fig. S1). This matter is further complicated by the lack of complete synchronicity between both phenotypes, across genotypes (Fig. 3 and fig. S1). Last, the longevity response to both a restricted diet and the reintroduction of a rich one may be contingent on the macronutrient composition of both (*3*, *5*). Earlier work diluted media reducing both carbohydrates and protein (*36*), in contrast to our method of reducing yeast concentration alone.

Genotypes will differ in their longevity reaction norm to diet, rendering it impossible to know a priori whether a certain dietary composition constitutes the exact optimal longevity-directed diet (*9*, *39*). Genetic variation in the response to DR, reported in rodents (*40*) and flies (*41*), might therefore not necessarily, or wholly, constitute variation in the physiological mechanisms that connect DR to ageing. We propose that our dietary phenotypes may also be contingent upon the direction and degree in which these diets deviate from the optimum, which may be one explanation for the dissimilarity of results observed in similar experiments. These considerations may also explain why the precise duration of DR is important, in line with the recent finding that the duration of starvation is critical in the life-span extension generated via intermittent fasting (*42*). In addition, larval diet, timing, and the order of how diets were fluctuated contributed to differential mortality observed when fluctuating diet (*43*). “Choice” experiments—where poor and rich diets are fed to flies in conjunction—result in heightened mortality, compared with continuous feeding (*44*). These effects are dependent on serotonin signaling, suggesting that the perceived rather than actual composition of food ingested modulates ageing (*45*).

In light of this, it is important to consider the renewed interest in intermittent fasting in both rodent and human studies (*1*, *46*). Studies in the previous century on rodents already demonstrated that inducing intermittent fasting, by feeding animals every other day or by other means, extends life span in a similar manner to caloric restriction [reviewed in (*47*)]. Two recent studies in mice suggest the same, although the effects are not as large as full caloric restriction (*48*), and outcomes for systemic ageing have been questioned (*49*). Human data on intermittent fasting are promising (*46*) and have potential application in specific diseases (*50*), but conclusive evidence from clinical trials is currently lacking (*51*). Our work now suggests that intermittent DR, dependent on its duration, can have negative consequences. These observations fit with the “refeeding syndrome”—a clinical condition that occurs at refeeding after a period of starvation (*52*). It remains to be determined which duration of starvation or DR would instigate such harmful physiological effects upon refeeding to the extent that it offsets its physiological benefits in humans. The responses we observe, however, are clearly not expected under the somatic maintenance hypothesis of DR, as flies appear to become maladapted to rich nutrient conditions under DR. In this vein, we appreciate that it has been suggested that naturalistic dietary conditions required to investigate DR are not appropriately mimicked in the laboratory and that DR itself is a laboratory-based artifact (*53*). Note that such a suggestion would preclude any inference from DR to our own species based on evolutionary arguments. That animals in the laboratory experience an unnaturally heightened nutritional state, not often available in the wild, is an idea not well supported. Careful studies have shown that wild and domestic mice have similar mass-adjusted metabolic rate, although they differ genetically and experience vastly different environments (*54*).

**Fig. 5:**
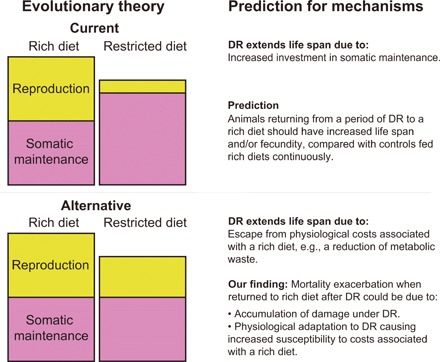
Schematic of the current, and alternative, hypotheses of DR. Reduced resource availability leading to increased investment toward somatic maintenance explains life-span extension under DR (see Fig. 1) in the most commonly supported current evolutionary theory. This increased investment may be absolute or relative to total resource availability. In our alternative model, based on the conclusions from the experiments we present here, the reduction in resource availability simply elicits a correlated reduction in available resources allocated toward reproductive output. The extension of life span observed under DR would then be a similarly passive response: an escape from unidentified costs incurred under a rich diet. These costs may be related to heightened metabolism or arising from direct insults of excessive protein intake. In addition, we propose restricted diets promote the accumulation of unknown costs, which are only observable upon resumption of a rich diet (not depicted here; see discussion). These hidden costs of DR would be responsible for the exacerbation of mortality observed when a rich diet is resumed. We suggest that these costs result from a period of physiological adaptation to a restricted diet, compensating for particular components of a rich diet. Such compensation on the DR diet, essentially maladapting the organisms to rich diet conditions, is directly contrary to current evolutionary theory that suggests investment in somatic maintenance occurs to survive to reap fitness benefits when resources are plentiful again.

At present, no mechanistic explanation is apparent, which explains the exacerbated mortality when flies return to a rich diet after a period of DR. We have excluded water balance (*26*), social effects (*27*), the microbiome (*28*), and sex-specific effects (*29*) as being wholly responsible for our observations. We therefore conclude that in conjunction with physiological costs associated with a rich diet, there are hidden costs associated with DR. These costs appear only when a rich diet is resumed after DR. The difference in mortality rates between our switching treatments (Fig. 2, B, C, E, and F) demonstrate a minimum period of acclimation to a restricted diet is necessary to generate the detectable costs of it. This suggests a physiological change at DR that makes animals more sensitive to rich diets, in direct contrast to predictions from evolutionary theory. Drawing from our observation of exacerbated mortality upon resumption of a rich diet—even when DR caused starvation—we suggest this exacerbation results from physiological adaptations that compensate for the lack of certain components within a restricted diet. Moreover, we observe these phenotypes across a range of genotypes with varying nutritional requirements—inferred from the existence of starvation in some lines on our experimental DR diet. This suggests that these effects will hold over a wide range of diet concentrations. Future experiments that gradually change diets over time, or titrate the difference in the diet required to recapitulate the observed phenotypes, could test this directly. We suggest that the physiological compensation that occurs at DR sensitizes animals to the physiological costs associated either with the elevated intake or metabolism of such a specific dietary component, leading to the exacerbation in mortality we observed. These effects could also originate more directly from compensation to nutrient restriction leading to an up-regulation of nutrient intake and metabolic recycling pathways, that upon resumption of the high-nutrient diet could lead to a detrimental influx of specific harmful dietary components or a higher flux through metabolic pathways (e.g., the generation of toxic by-products). These same, otherwise hidden, mechanisms might also underlie why animals fed rich diets continuously are shorter lived than those on DR: as an escape from costs associated with the intake or metabolism of a (or several) dietary component(s) (Fig. 5). This paradigm also explains why flies can rapidly and repeatedly lower their mortality in response to DR.

A recent reappreciation of the evolutionary biology of DR (and molecular nutrient-sensing pathways) suggests that phenotypic plasticity is at the core of the evolutionary explanation of DR (*19*). We find that flies are highly plastic in modulating their reproduction to dietary conditions. Death through depletion of bodily resources to use in reproduction would not be optimal if the animal expects resources to increase at some point in their lives. Such phenotypic plasticity does not directly explain, however, why animals on DR live longer than their fully fed counterparts, unless phenotypic plasticity itself or the act of reproduction carries specific costs. We know from careful experiments in model organisms that the effects of DR are largely independent of reproduction (*9*, *55*–*57*). This therefore suggests that the reduction in reproduction with DR is a correlated phenotypic response that is not causative in the DR longevity response. Such observations fit with recent elegant experiments showing that artificial selection for reproduction during DR does not affect the DR longevity response (*20*).

All current evidence to date suggests that uptake of the macronutrient protein is responsible for the effects of diet on longevity (*3*–*5*). We suggest that DR’s effect on longevity is not via increased investment in somatic maintenance, but the result from a forced escape from the intrinsically harmful effects of dietary protein. The reason why animals would still choose to eat or absorb intrinsically harmful components, such as protein from their diets, is most likely for its use in reproduction in both sexes (*3*, *5*, *16*). The specific physiological mechanisms that underlie these costs lie at the heart of DR’s life-span–extending capacities. Our identification of previously unidentified dietary phenotypes in the fly that expose these otherwise hidden costs could prove a powerful new experimental phenotype for the mechanistic study of DR. We suggest that the quest to identify the mechanisms of DR will be aided by acceptance that somatic maintenance is not necessarily responsible for the life extension seen under DR.

## MATERIALS AND METHODS

### Fly husbandry

Wild-type inbred isofemale flies from the *D. melanogaster* genetic reference panel (*58*) were acquired from the Bloomington Stock Center and the laboratory of Bart Deplancke (EPFL). Flies were cultured on rich media [8% autolyzed yeast, 13% table sugar, 6% cornmeal, 1% agar, and nipagin 0.225% (w/v)] with bottles for growing and mating, containing an additional 0.4% (v/v) propanoic acid. For life-span experiments, adult flies were subsequently provided with either the same rich media or a restricted media (2% autolyzed yeast) in vials. These dietary concentrations are neither particularly rich nor restricted in comparison to published work (*36*, *57*). Diets remain difficult to compare between studies as ingredients and fly media preparation differ between laboratories. Our rich and restricted diets induce consistent life-span differentials. Moreover, recent work carried out using a wider range of diets suggests that our diets are in the area of largest response for most genotypes (*59*). Restricted media retained the composition of all other media components, given the dietary protein axis is the main life-span determinant in flies (*3*, *5*). Cooked fly medium was kept for a maximum of 2 weeks at 4° to 6°C and was warmed to 25°C before use.

### Experimental mortality protocol and demography cages

Flies were expanded in bottles (*Drosophila* PP Flask Square Bottom; Flystuff) on a rich diet. Experimental flies were grown in bottles (incubated at 25°C) sprinkled with granulated live yeast, in which 12 females and 2 males had been egg laying for a period of ~60 hours. Bottles were sprinkled with water, daily, if media appeared dry until pupation began. Upon eclosion, the adult F1 generation was transferred, daily to generate age-matched cohorts, to mating bottles for 48 hours before being sorted under light CO_2_ anesthesia (Flystuff Flowbuddy; <5 liters/min) and transferred to purpose-built demography cages (*37*). Life-span experiments were carried out in a climate-controlled room (12:12 light/dark cycle 25°C and 50 to 60% relative humidity). Cages contained between 100 and 125 females each; the number of cages was treatment dependent. All flies were kept on rich media until age 3 to 6 days, whereupon they were divided between the dietary treatments. Individual life span was determined from the time when the individual entered the experimental cage (at 2 days of age) until death or censoring. A census of flies was taken every other day: Dead flies were counted and removed, and fresh medium was provided at this time. Flies that were alive but stuck to the side of the vial, escaped flies, and individuals affixed to the food (~10.5% of deaths) were right censored.

### Fecundity

A subsection of fly feeding vials were imaged and analyzed using QuantiFly (*60*) to determine relative amounts of egg laying.

### Dietary regimes

Two main temporal dietary regimes were imposed on several genotypes of mainly female flies using two diets, restricted (DR, 2% yeast) and rich (8% yeast), with controls of continuous exposure to these diets.

1) To test whether a prolonged period of DR resulted in superior survival and reproduction when conditions improved, flies were exposed to continuous restricted diet that was switched to a rich diet at ~45 to 60% survival of the continuous rich-diet group (long-switch). All flies of the same genotype were switched on the same day, irrespective of eclosion date.

2) We further tested whether short bouts of DR had similar effects, which also allowed us to test whether effects observed in the long-switch regime were exclusive to older flies. In these diets, flies were repeatedly switched between restricted and rich diets at 4-day intervals (4-day switch). By starting half of the experimental cohort on restricted or rich diets, current dietary treatments were mirrored and balanced across the cohort.

These experiments were performed on DGRP-195 at high sample size (*N* = 14,102). Subsequently, to test whether these effects were general, these experiments were expanded to a panel of DGRP lines (DGRP-105, 136, 195, 217, 239, 335, 362, 441, 705, 707, and 853) in one large experiment of *N* = 37,897. Several other parts of the experiments (see below) were run separately (for specific grouping, see the Supplementary Materials). Dietary treatments were balanced for age. From this experiment, fecundity estimates were also taken from feeding vials on four consecutive scoring days (for 4-day switch and continuous treatments) and one scoring day before and after the dietary switch (for long-switch and continuous rich treatment).

### Supplementary dietary regimes

We tested a range of other dietary regimes to test specific hypotheses, alongside the treatments listed above, using line DGRP-195. (i) We tested whether DR could instantly reduce mortality by imposing a short duration (4 days) of DR in late life, sensu Mair *et al.* (*36*), before returning to a rich diet (short reverse-switch). (ii) We increased the frequency of the dietary switch to 2 days (2-day switch) to investigate the length of DR necessary for the observed phenotypes and (iii) further changed the ratio of the time spent on either diet, 2 days of either rich or restricted diet to 4 days of the reverse (4-to-2–day switch).

### Tests of specific hypotheses: Microbiome, water balance, sex, and social effects

We tested whether the dietary phenotypes observed were due to four potential previously suggested confounding factors: (i) microbiome (*28*), (ii) water balance (*26*), (iii) social effects (*27*), and (iv) sex differences in the DR response (*29*). These were confirmed not to interfere with the observed phenotype (see Results). DGRP-195 was used exclusively for these experiments under the continuous restricted, continuous rich, and long-switch diets. As our original dietary switch genotype, we reasoned that exclusion of these potentially confounding variables in this one genetic line would preclude them from being principal causative agents. Note, however, that this means we cannot strictly exclude that in other genotypes these confounding effects are more important. (i) We assessed whether disruption of the gut microbiome was responsible for the mortality phenotype observed by wholesale abating the microbiome. Flies were provided media upon which an array of broad-spectrum antibiotics [50 μl of a stock solution, composed of ampicillin (100 μg/ml), vancomycin (50 μg/ml), neomycin (100 μg/ml), and metronidazole (100 μg/ml)] were pipetted and left for 24 hours. We assumed dissolution incorporation in the top 1 ml of food. Antibiotic treatment began 4 days before dietary switch treatments and concluded 8 days thereafter. Ablation of the microbiome was confirmed by whole-fly homogenization (age, 20 days; 8 days after antibiotic treatment) and growth of solution on MRS agar plates (Oxoid; see fig. S7). Individuals (six control and six antibiotic treated) were removed from cages containing a continuous restricted diet, washed in ethanol, and rinsed in phosphate-buffered saline (PBS) (Gibco). Homogenization took place in 500 μl of PBS, and solute was transferred to a 96-well plate for 1:10 serial dilutions. Dilutions were spotted on plates with and without antibiotic (500 μl of stock solution) and incubated at 25°C for 72 hours. Plates were coated with parafilm to mimic anoxic conditions. ii) Flies were provided with ~1 cm^3^ portion of water-agar (2%, w/v) accompanying media in vials to eliminate desiccation as a proximal cause. Water-agar supplementation began at age 4 and continued throughout the flies’ full life course. (iii) Social effects were excluded by housing flies individually in vials. These flies were taken from experimental cages and put on the experimental diets at the dietary switch. (iv) Males were assessed for mortality in the 4-day switch dietary regime.

### Experimental batches

All demography experiments contained the relevant controls, grown, and assayed for mortality at the same time. Where data are plotted in a single figure, this constitutes results gathered from a batch of flies at the same chronological time.

### Data analysis

Mixed Cox proportional hazard models were used that included “cage” as random term to correct for uncertainty of pseudoreplicated effects within demography cages (*61*). We used interval-based models that used time-dependent covariates to estimate the differential mortality risks associated with diet (and with time spent on a diet, after diets changed), as imposed in the different dietary regimes. These models allow a statistical association, within the Cox proportional hazard risk, with the current state (e.g., diet) and mortality. Flies in the long-switch dietary regime were also analyzed in a state-dependent manner, coding for long-switch only when this state change occurred. Repeated switching regimes were considered lifelong treatments and tested in interaction with the state variable diet. Each model used continuous rich food and DGRP-195 as reference category, except if otherwise stated.

Interactions between dietary regime, diet, and genotype were fitted to test for differential effects of diet on mortality depending on the regime it was provided. Additional specific tests of coefficients were provided that combine the single and interaction term (in a *z* test, using the maximum SE of the factor compared) to test how mortality risk was changing compared with specific reference categories of interest (e.g., compared with continuous DR). For comparisons between genotypes, we report full models including all data and models fitted within each genotype separately. The latter corrects for deviations in proportionality of hazards between the genotypes. Qualitative conclusions remain similar, and formal tests for proportionality of hazards are not available for mixed effects Cox regressions. Models without a time-dependent covariate for diet were also run to compare overall longevity differences as a result of alternating exposure to DR (2-day switch, 4-day switch, and their combination). These models therefore test the integrated effect on mortality, disregarding any within–dietary treatment diet effects. Coefficients are reported as logged hazards with significance based on *z* tests. Right censoring was included, as indicated above.

Egg laying was analyzed using a linear (mixed) model using “cage” as random term and fitting age as a noncontinuous factor in the analysis. Estimates from models are presented (effects of dietary regime) as well as model comparisons using log-likelihood comparison with chi-square to test overall effects of genotype. Effects of different dietary regimes were estimated within the same model. Comparisons of genotypic effects were performed for each different dietary regime separately compared with continuous treatment, as not to conflate genetic variance across different categories with each other.

## Supplementary Material

http://advances.sciencemag.org/cgi/content/full/6/8/eaay3047/DC1

Download PDF

The hidden costs of dietary restriction: Implications for its evolutionary and mechanistic origins

## SUPPLEMENTARY MATERIALS

Supplementary material for this article is available at http://advances.sciencemag.org/cgi/content/full/6/8/eaay3047/DC1 Fig. S1. Four-day switch treatment in a panel of 11 DGRP genotypes. Fig. S2. Fecundity analysis of long-switch treatment from three DGRP genotypes. Fig. S3. Fecundity analysis of 4-day switch treatment from 10 DGRP genotypes. Fig. S4. Four-day switch treatment of DGRP-195 males. Fig. S5. Antibiotic long-switch treatment of DGRP-195. Fig. S6. Water-supplemented long-switch treatment of DGRP-195. Fig. S7. Confirmation of ablation of microbiome. Table S1. Effect of dietary regimes on interval-based log hazard ratios of mortality in DGRP-195. Table S2. Effect of dietary regimes on longevity in DGRP-195. Table S3. Time-dependent effect of mortality increase induced by a long-switch from reduced to rich diets in DGRP-195. Table S4. Time-dependent effect of mortality increase induced by a 4-day switch in DGRP-195. Table S5. Effect of asymmetrical dietary regimes on mortality in DGRP-195. Table S6. Effect of asymmetrical dietary regimes on longevity in DGRP-195. Table S7. Mortality increases in response to a rich diet after a period of DR (long-switch) across a panel of 11 DGRP lines (195 is reference). Table S8. Models run within each genotype testing for increases in response to a rich diet after a period of DR (long-switch). Table S9. Effect of alternating DR and rich diets every 4 days (4-day switch) on longevity across 11 DGRP lines (195 is reference). Table S10. Effect of alternating DR and rich diets every 4 days (4 day switch) on mortality at each diet, across 11 DGRP lines (195 is reference). Table S11. Interval models run within each genotype testing for differential effects of diet in the 4-day switch dietary regime. Table S12. Models run within each genotype testing for increases in response to a rich diet after a period of DR (long-switch) but within lines that showed starvation only and with DR as reference category. Table S13. Interval models run within each genotype testing for differential effects of diet in the 4-day switch dietary regime but within lines that showed starvation only and with DR as reference category. Table S14. Linear model of estimates of (log-transformed) fecundity (from Quantifly) in the long-switch dietary treatment. Table S15. Linear model of estimates of (log-transformed) fecundity (from Quantifly), corrected for number of flies in the cage, in the long-switch dietary treatment. Table S16. Mixed model (correcting for cage) of estimates of (log-transformed) fecundity (from Quantifly) in the 4-day switching paradigm. Table S17. Mixed model (correcting for cage) of estimates of (log-transformed) fecundity (from Quantifly), corrected for number of flies in the cage, in the 4-day switching paradigm. Table S18. Effect of returning to a rich diet after a period of DR (long-switch) after ablation of the microbiome (antibiotics on rich diet is reference). Table S19. Effect of returning to a rich diet after a period of DR (long-switch) with supplementation of water. Table S20. Effect of returning to a rich diet after a period of DR (long-switch) with flies in isolation in vials. Table S21. Effect of switching from DR to rich food every 4 days (4-day switch) in males.

## Appendix

View/request a protocol for this paper from *Bio-protocol*.
