# Supplementary material for: The hidden costs of dietary restriction: Implications for its evolutionary and mechanistic origins
Source: Sci Adv. 2020 Feb 21;6(8):eaay3047. doi: 10.1126/sciadv.aay3047 (PMC7034997; doi:10.1126/sciadv.aay3047)
Supplement: http://advances.sciencemag.org/cgi/content/full/6/8/eaay3047/DC1 [file supp_6_8_eaay3047__index.html]

Science Advances | Science AdvancesAAASSearchScience AdvancesMenu

## Supplementary Materials

**This PDF file includes:**

- Fig. S1. Four-day switch treatment in a panel of 11 DGRP genotypes.
- Fig. S2. Fecundity analysis of long-switch treatment from three DGRP genotypes.
- Fig. S3. Fecundity analysis of 4-day switch treatment from 10 DGRP genotypes.
- Fig. S4. Four-day switch treatment of DGRP-195 males.
- Fig. S5. Antibiotic long-switch treatment of DGRP-195.
- Fig. S6. Water-supplemented long-switch treatment of DGRP-195.
- Fig. S7. Confirmation of ablation of microbiome.
- Table S1. Effect of dietary regimes on interval-based log hazard ratios of mortality in DGRP-195.
- Table S2. Effect of dietary regimes on longevity in DGRP-195.
- Table S3. Time-dependent effect of mortality increase induced by a long-switch from reduced to rich diets in DGRP-195.
- Table S4. Time-dependent effect of mortality increase induced by a 4-day switch in DGRP-195.
- Table S5. Effect of asymmetrical dietary regimes on mortality in DGRP-195.
- Table S6. Effect of asymmetrical dietary regimes on longevity in DGRP-195.
- Table S7. Mortality increases in response to a rich diet after a period of DR (long-switch) across a panel of 11 DGRP lines (195 is reference).
- Table S8. Models run within each genotype testing for increases in response to a rich diet after a period of DR (long-switch).
- Table S9. Effect of alternating DR and rich diets every 4 days (4-day switch) on longevity across 11 DGRP lines (195 is reference).
- Table S10. Effect of alternating DR and rich diets every 4 days (4 day switch) on mortality at each diet, across 11 DGRP lines (195 is reference).
- Table S11. Interval models run within each genotype testing for differential effects of diet in the 4-day switch dietary regime.
- Table S12. Models run within each genotype testing for increases in response to a rich diet after a period of DR (long-switch) but within lines that showed starvation only and with DR as reference category.
- Table S13. Interval models run within each genotype testing for differential effects of diet in the 4-day switch dietary regime but within lines that showed starvation only and with DR as reference category.
- Table S14. Linear model of estimates of (log-transformed) fecundity (from Quantifly) in the long-switch dietary treatment.
- Table S15. Linear model of estimates of (log-transformed) fecundity (from Quantifly), corrected for number of flies in the cage, in the long-switch dietary treatment.
- Table S16. Mixed model (correcting for cage) of estimates of (log-transformed) fecundity (from Quantifly) in the 4-day switching paradigm.
- Table S17. Mixed model (correcting for cage) of estimates of (log-transformed) fecundity (from Quantifly), corrected for number of flies in the cage, in the 4-day switching paradigm.
- Table S18. Effect of returning to a rich diet after a period of DR (long-switch) after ablation of the microbiome (antibiotics on rich diet is reference).
- Table S19. Effect of returning to a rich diet after a period of DR (long-switch) with supplementation of water.
- Table S20. Effect of returning to a rich diet after a period of DR (long-switch) with flies in isolation in vials.
- Table S21. Effect of switching from DR to rich food every 4 days (4-day switch) in males.

Download PDF

**Files in this Data Supplement:**

- Adobe PDF - aay3047\_SM.pdf
