# Supplementary material for: The hidden costs of dietary restriction: Implications for its evolutionary and mechanistic origins
Source: Sci Adv. 2020 Feb 21;6(8):eaay3047. doi: 10.1126/sciadv.aay3047 (PMC7034997; doi:10.1126/sciadv.aay3047)
Supplement: Download PDF [file aay3047_SM.pdf]

Science Advances
AAAS

advances.sciencemag.org/cgi/content/full/6/8/eaay3047/DC1

Supplementary Materials for

The hidden costs of dietary restriction: Implications for its evolutionary and mechanistic origins

Andrew W. McCracken, Gracie Adams, Laura Hartshorne, Marc Tatar, Mirre J. P. Simons*

*Corresponding author. Email: m.simons@sheffield.ac.uk

Published 21 February 2020, *Sci. Adv.* **6**, eaay3047 (2020)
DOI: 10.1126/sciadv.aay3047

This PDF file includes:

Fig. S1. Four-day switch treatment in a panel of 11 DGRP genotypes.
Fig. S2. Fecundity analysis of long-switch treatment from three DGRP genotypes.
Fig. S3. Fecundity analysis of 4-day switch treatment from 10 DGRP genotypes.
Fig. S4. Four-day switch treatment of DGRP-195 males.
Fig. S5. Antibiotic long-switch treatment of DGRP-195.
Fig. S6. Water-supplemented long-switch treatment of DGRP-195.
Fig. S7. Confirmation of ablation of microbiome.
Table S1. Effect of dietary regimes on interval-based log hazard ratios of mortality in DGRP-195.
Table S2. Effect of dietary regimes on longevity in DGRP-195.
Table S3. Time-dependent effect of mortality increase induced by a long-switch from reduced to rich diets in DGRP-195.
Table S4. Time-dependent effect of mortality increase induced by a 4-day switch in DGRP-195.
Table S5. Effect of asymmetrical dietary regimes on mortality in DGRP-195.
Table S6. Effect of asymmetrical dietary regimes on longevity in DGRP-195.
Table S7. Mortality increases in response to a rich diet after a period of DR (long-switch) across a panel of 11 DGRP lines (195 is reference).
Table S8. Models run within each genotype testing for increases in response to a rich diet after a period of DR (long-switch).
Table S9. Effect of alternating DR and rich diets every 4 days (4-day switch) on longevity across 11 DGRP lines (195 is reference).
Table S10. Effect of alternating DR and rich diets every 4 days (4 day switch) on mortality at each diet, across 11 DGRP lines (195 is reference).
Table S11. Interval models run within each genotype testing for differential effects of diet in the 4-day switch dietary regime.
Table S12. Models run within each genotype testing for increases in response to a rich diet after a period of DR (long-switch) but within lines that showed starvation only and with DR as reference category.

Table S13. Interval models run within each genotype testing for differential effects of diet in the 4-day switch dietary regime but within lines that showed starvation only and with DR as reference category.
Table S14. Linear model of estimates of (log-transformed) fecundity (from Quantifly) in the long-switch dietary treatment.
Table S15. Linear model of estimates of (log-transformed) fecundity (from Quantifly), corrected for number of flies in the cage, in the long-switch dietary treatment.
Table S16. Mixed model (correcting for cage) of estimates of (log-transformed) fecundity (from Quantifly) in the 4-day switching paradigm.
Table S17. Mixed model (correcting for cage) of estimates of (log-transformed) fecundity (from Quantifly), corrected for number of flies in the cage, in the 4-day switching paradigm.
Table S18. Effect of returning to a rich diet after a period of DR (long-switch) after ablation of the microbiome (antibiotics on rich diet is reference).
Table S19. Effect of returning to a rich diet after a period of DR (long-switch) with supplementation of water.
Table S20. Effect of returning to a rich diet after a period of DR (long-switch) with flies in isolation in vials.
Table S21. Effect of switching from DR to rich food every 4 days (4-day switch) in males.

Supplementary Figures and Tables

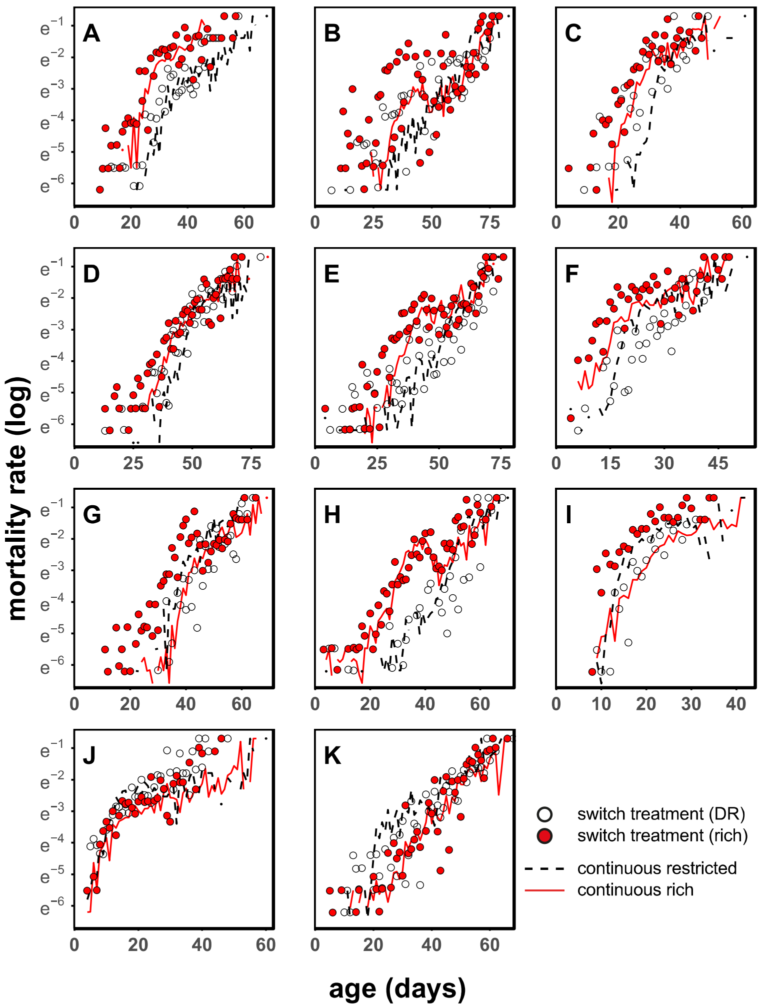

Fig. S1. Four-day switch treatment in a panel of 11 DGRP genotypes. A – 195; B – 105; C – 217; D – 441; E – 705; F – 707; G – 136; H – 362; I – 239; J – 335; K – 853. Continuous rich, and restricted treatments plotted as lines (solid red and dashed black, respectively). Switch treatments plotted as points (white and red). The exacerbation of mortality due to switch phenotypes is observable as the difference between mortality at continuous rich diet (red line), and mortality of switch treatment when on a rich diet (red points). N = 29,740 females total; ~2,725 females per genotype; 13,375 for continuous rich treatments, and ~8,170 for continuous rich and 4-day switch treatments. Dietary switch for 4-day switch treatment group occurred every 4 days, and was mirrored at each time point. Continuous rich and restricted treatments are twinned with long switch treatment experiment (Fig. 2). All panels contain daily time-points, as in Fig.2.

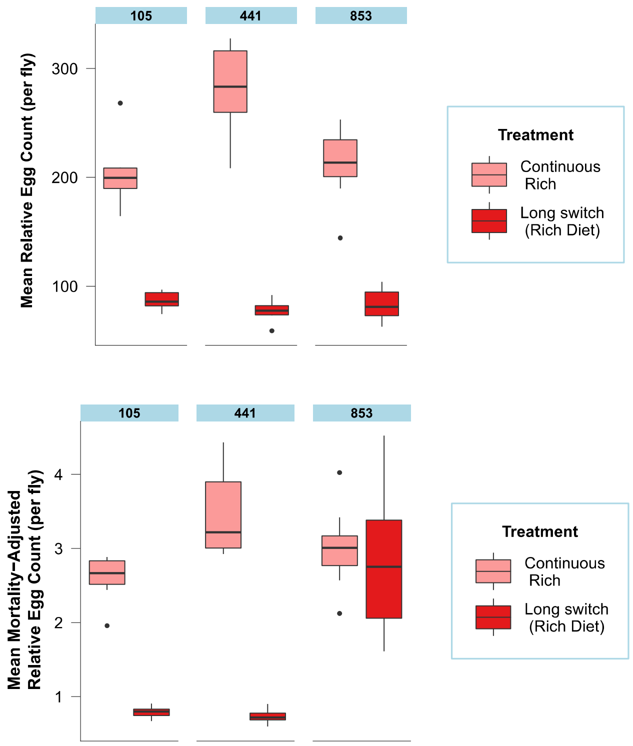

Fig. S2. Fecundity analysis of long-switch treatment from three DGRP genotypes. No compensation via fecundity for reduced lifespans in switch treatment. Raw (above) and mortality corrected (below) egg counts of DGRP-105; 441; 853 from long switch treatment experiment (Fig. 2). Counts generated using QuantiFly software. Counts are relative, but directly comparable. Flies assayed between age 44-47 days, with boxplots (median, with the box depicting a quartile each way, and whiskers showing the range; outliers plotted as dots) aggregating totals. Each cage was assayed once, on the first scoring day post dietary switch. Mortality corrected counts (below) generated by dividing raw counts, by N flies remaining in cage at the time of assaying. N = on average, 7 cages assayed, per treatment, per genotype.

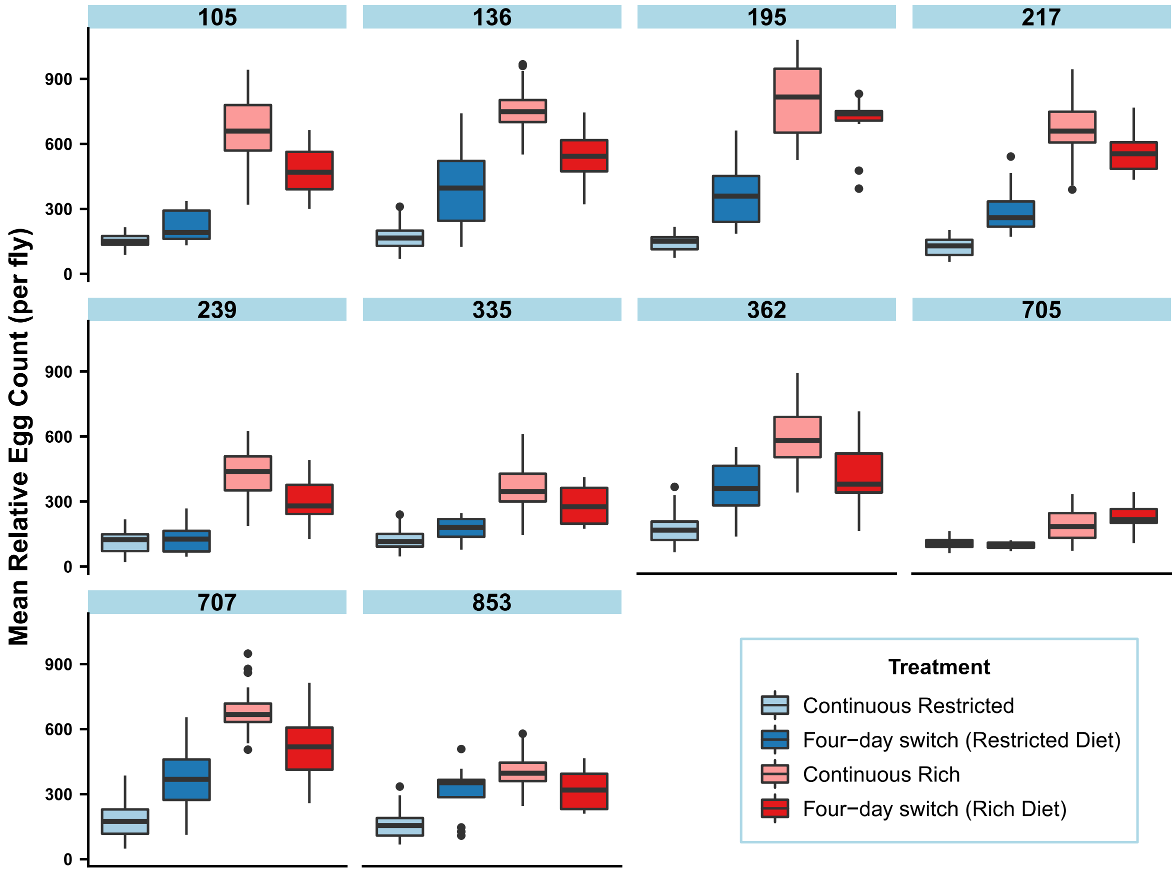
Mean Relative Egg Count (per fly)
105
136
195
217
239
335
362
705
707
853
0
300
600
900
Treatment
Continuous Restricted
Four−day switch (Restricted Diet)
Continuous Rich
Four−day switch (Rich Diet)

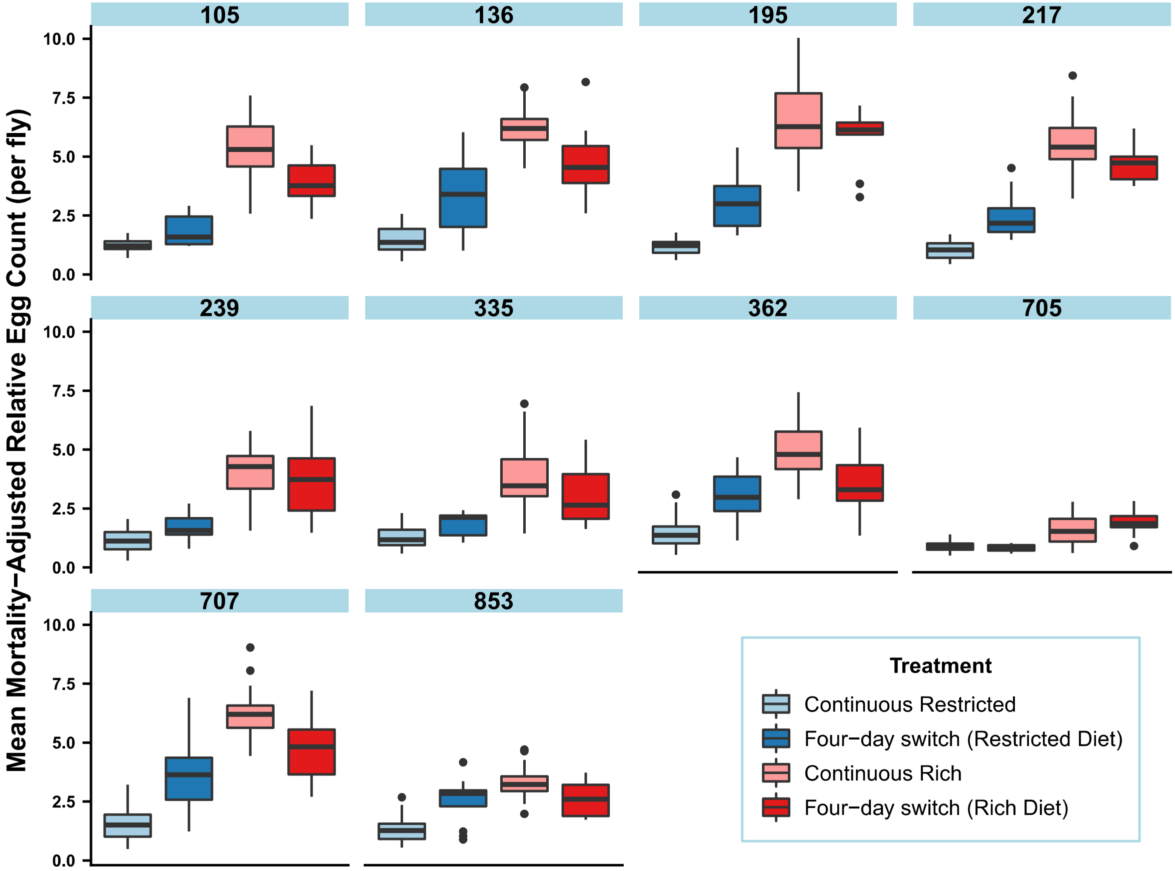
Mean Mortality−Adjusted Relative Egg Count (per fly)
105
136
195
217
239
335
362
705
707
853
0.0
2.5
5.0
7.5
10.0
Treatment
Continuous Restricted
Four−day switch (Restricted Diet)
Continuous Rich
Four−day switch (Rich Diet)

Fig. S3. Fecundity analysis of 4-day switch treatment from 10 DGRP genotypes. No compensation via fecundity for reduced lifespans in switch treatment. Raw (above) and mortality corrected (below) egg counts of DGRP-105; 136; 195; 217; 239; 335; 362; 705; 707; 853 from long switch treatment experiment (Fig. 2). Counts generated using QuantiFly software. Counts are relative, but directly comparable. Flies assayed between age 8-21 days, with boxplots aggregating totals (median, with the box depicting a quartile each way, and whiskers showing the range; outliers plotted as dots). Each cage was assayed on 4 consecutive scoring days. Mortality corrected counts (below) generated by dividing raw counts, by N flies remaining in cage at the time of assaying. N = on average, 7 cages assayed, per treatment, per genotype.

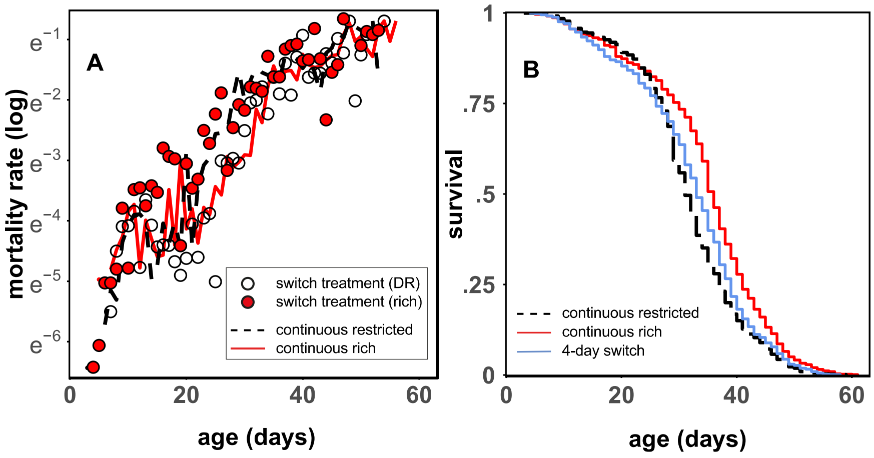

Fig. S4. Four-day switch treatment of DGRP-195 males. A – 4-day switch mortality; B – 4-day switch survival. Muted response to 4-day switch treatment in males. Rich diet in the 4-day switch increased mortality compared to continuously rich fed flies. Continuous rich, and restricted treatments plotted as lines (solid red and dashed black, respectively). Switch treatment plotted as points (white and red). The exacerbation of mortality due to switch phenotypes is observable as the difference between mortality at continuous rich diet (red line), and mortality of switch treatment when on a rich diet (red points). N = 4,429 total; ~1,475 per treatment. Dietary switch for 4-day switch treatment group occurred every 4 days, and was mirrored at each time point. Both panels contain daily time-points, as in Fig.2.

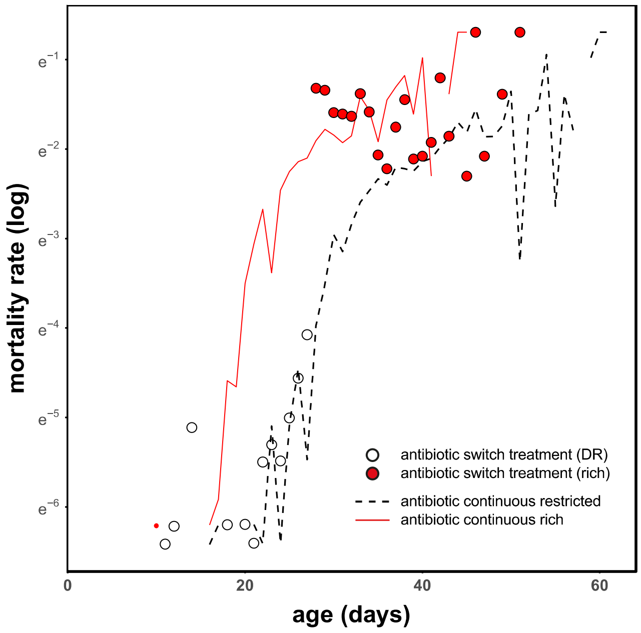

Fig. S5. Antibiotic long-switch treatment of DGRP-195. Long switch phenotype independent of antibiotic treatment. Antibiotic treatment took place in all treatments four days prior to dietary switch, and concluded eight days thereafter. Continuous rich, and restricted treatments plotted as lines (solid red and dashed black, respectively). Switch treatment plotted as points (white and red). The exacerbation of mortality due to switch phenotypes is observable as the difference between mortality at continuous rich diet (red line), and mortality of switch treatment when on a rich diet (red points). N = 2,605 females total; ~870 per treatment. (See Fig. S7 for confirmation of ablation of microbiome). Figure contains daily time-points, as in Fig.2.

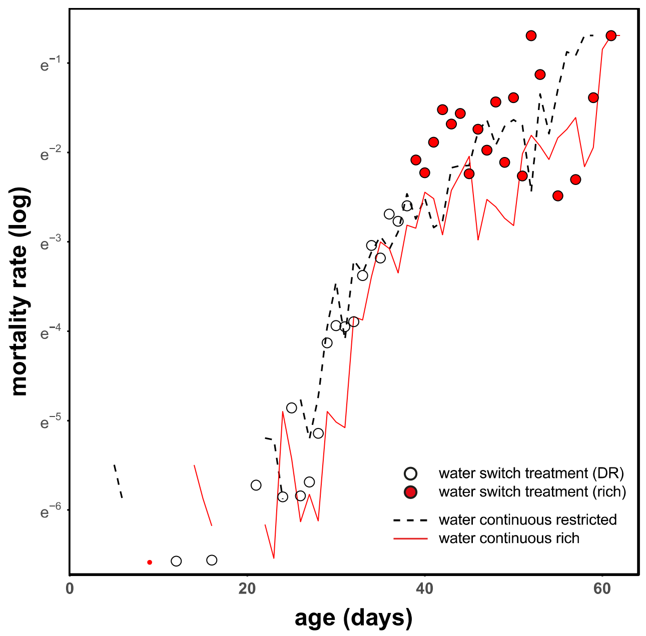

Fig. S6. Water-supplemented long-switch treatment of DGRP-195. Long switch phenotype independent of water supplementation. Water supplementation took place in all treatments throughout life of the cage. Continuous rich, and restricted treatments plotted as lines (solid red and dashed black, respectively). Switch treatment plotted as points (white and red). The exacerbation of mortality due to switch phenotypes is observable as the difference between mortality at continuous rich diet (red line), and mortality of switch treatment when on a rich diet (red points). N = 2,562 females total; ~850 per treatment. NB water supplementation did change the response to DR. This effect was followed up with an experiment containing five different genotypes across a range of diets, with only a shift in reaction norm detected (manuscript in preparation). DR is not explained by dehydration, as is sometimes suggested, nor is the long switch phenotype. Figure contains daily time-points, as in Fig.2.

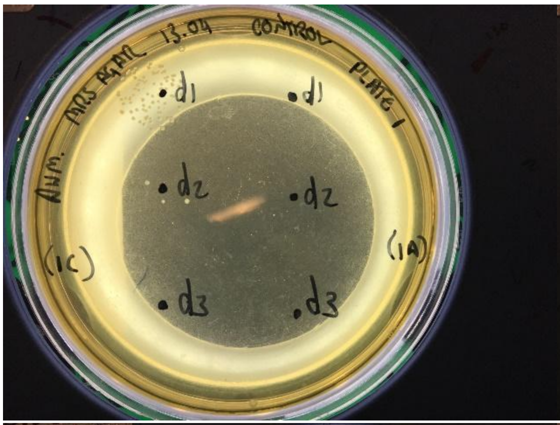
AWM. MRS AGAR 13.04 CONTROL PLATE 1
d1 d1
d2 d2
(1C) (1A)
d3 d3

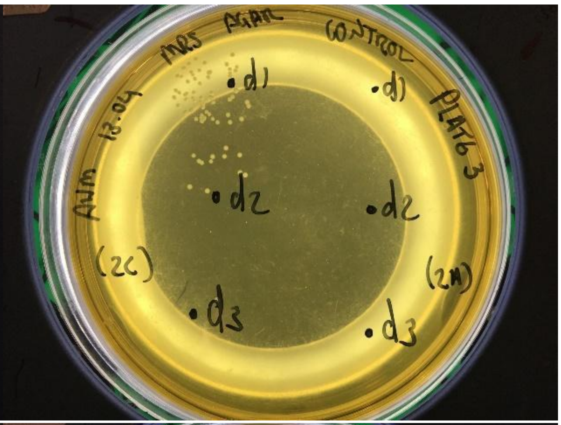
AWM 13.04 MRS AGAR CONTROL PLATE 3
d1 d1
d2 d2
(2C) (2A)
d3 d3

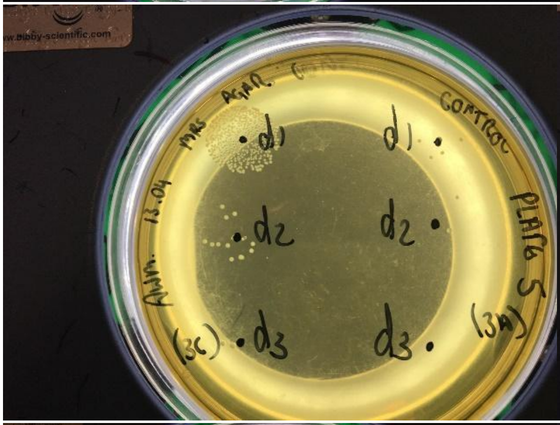
AWM. 13.04 MRS AGAR CONTROL PLATE 5
d1 d1
d2 d2
(3C) d3 d3 (3A)

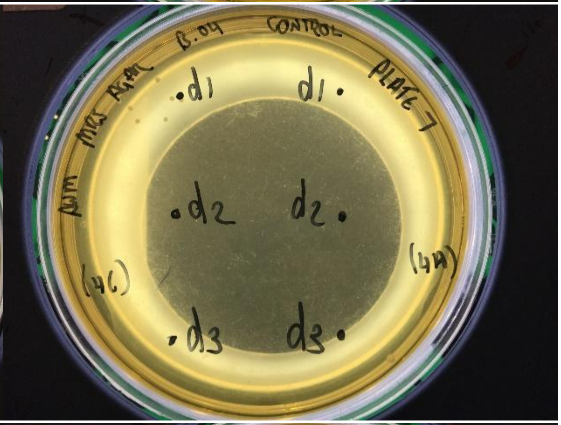
AWM MRS AGAR 13.04 CONTROL PLATE 7
d1 d1
d2 d2
(4C) (4A)
d3 d3

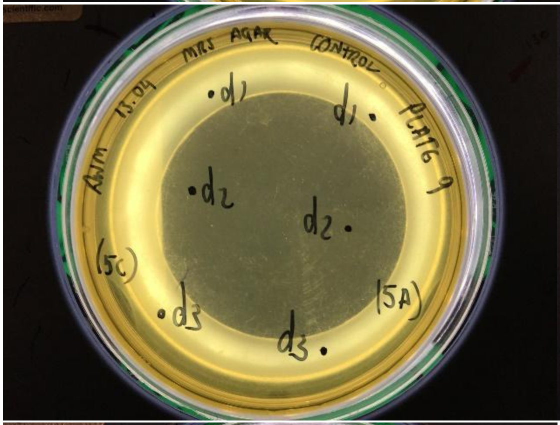
AWM 13.04 MRS AGAR CONTROL PLATE 9
d1 d1
d2 d2
(5C) (5A)
d3 d3

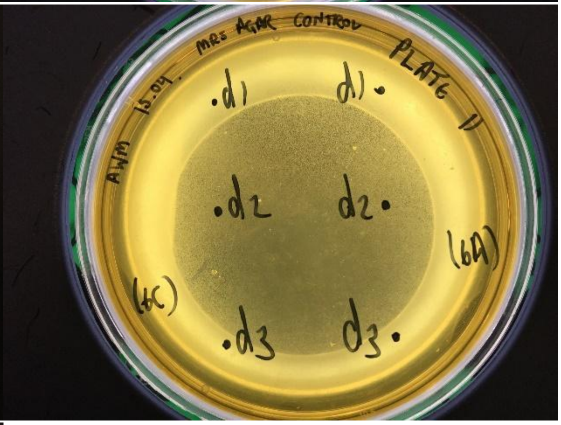
AWM 13.04 MRS AGAR CONTROL PLATE 11
d1 d1
d2 d2
(6A) (6C)
d3 d3

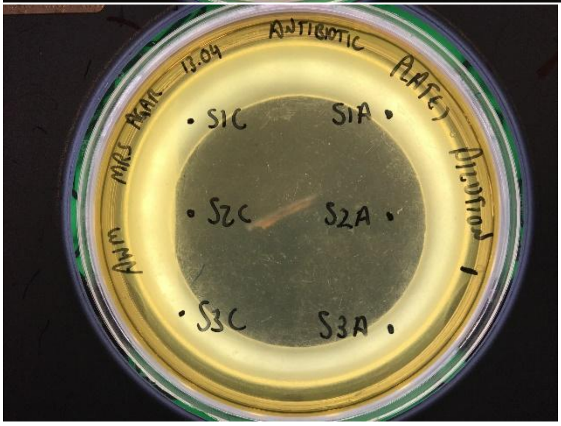
AWM MRS AGAR 13.04 ANTIBIOTIC PLATE DILUTION 1
S1C S1A
S2C S2A
S3C S3A

Sample	Colonies at dilution 1	Colonies at dilution 2	Colonies at dilution 3	Colonies at antibiotic plate dilutions
1 (control)	75	3	0	0
2 (control)	50	12	1	0
3 (control)	250	12	2	0
4 (control)	8	0	0	NA
5 (control)	0	0	0	NA
6 (control)	1	0	0	NA
7 (antibiotic treated)	0	0	0	0
8 (antibiotic treated)	0	0	0	0
9 (antibiotic treated)	6	2	0	0
10 (antibiotic treated)	0	0	0	NA
11 (antibiotic treated)	0	0	0	NA
12 (antibiotic treated)	0	0	0	NA

Fig. S7. Confirmation of ablation of microbiome. Images of bacterial colonies visible on MRS agar plates (above) and estimated colony count (below). Twelve samples ceded from control, or antibiotic-treated cages. Lysate was diluted post-homogenisation and grown on control, or antibiotic-treated plates. No growth visible under antibiotic treated plate conditions. 98.4% reduction of total microbiota observed at dilution 1. 92.5% reduction of total microbiota observed at dilution 2.

Table S1. Effect of dietary regimes on interval-based log hazard ratios of mortality in DGRP-195.

	Full Model				Versus continuous DR		
coefficient	estimate	exp (estimate)	s.e.	p	estimate	exp (estimate)	p
DR	-1.179	0.308	0.047	**<0.001**			
long switch	1.310	3.705	0.055	**<0.001**			
4-day switch	0.858	2.358	0.062	**<0.001**			
2-day switch	0.125	1.133	0.063	**0.047**			
short reverse-switch	0.272	1.312	0.051	**<0.001**			
4-day switch * DR	-0.409	0.665	0.085	**<0.001**	0.449	1.567	**<0.001**
2-day switch * DR	0.255	1.291	0.074	**0.001**	0.380	1.462	**<0.001**
short switch * DR	-0.466	0.627	0.116	**<0.001**	-0.195	0.823	0.092

Table S2. Effect of dietary regimes on longevity in DGRP-195.

	Full Model			
coefficient	estimate	exp (estimate)	s.e.	p
DR	-1.136	0.321	0.058	**<0.001**
2-day switch	-0.224	0.799	0.064	**<0.001**
4-day switch	0.386	1.471	0.064	**<0.001**

Table S3. Time-dependent effect of mortality increase induced by a long-switch from reduced to rich diets in DGRP-195.

	Full Model			
coefficient	estimate	exp (estimate)	s.e.	p
day 2	1.629	5.101	0.085	**<0.001**
day 4	0.847	2.334	0.093	**<0.001**
day 6	0.452	1.572	0.121	**<0.001**
day 8	-0.045	0.956	0.216	0.84
day 10	-0.226	0.798	0.369	0.54
day 12	-0.077	0.926	0.601	0.9
> day 14	-1.038	0.354	1.066	0.33

Table S4. Time-dependent effect of mortality increase induced by a 4-day switch in DGRP-195.

	Full Model			
coefficient	estimate	exp (estimate)	s.e.	p
DR	-1.303	0.272	0.094	**<0.001**
2nd interval	0.227	1.255	0.055	**<0.001**
2nd interval * DR	-0.715	0.489	0.143	**<0.001**

Table S5. Effect of asymmetrical dietary regimes on mortality in DGRP-195.

	Full Model				Versus continuous DR		
coefficient	estimate	exp (estimate)	s.e.	p	estimate	exp (estimate)	p
DR	-1.684	0.186	0.087	**<0.001**			
4d-DR|2d-Rich	-0.209	0.811	0.097	**0.032**			
2d-DR|4d-Rich	-0.157	0.854	0.095	0.097			
4d-DR|2d-Rich * DR	0.458	1.581	0.113	**<0.001**	0.249	1.283	**0.027**
2d-DR|4d-Rich * DR	0.929	2.531	0.124	**<0.001**	0.771	2.163	**<0.001**

Table S6. Effect of asymmetrical dietary regimes on longevity in DGRP-195.

	Full Model			
coefficient	estimate	exp (estimate)	s.e.	p
DR	-1.692	0.184	0.087	**<0.001**
4d-DR|2d-Rich	-0.805	0.447	0.095	**<0.001**
2d-DR|4d-Rich	-0.343	0.710	0.094	**<0.001**

Table S7. Mortality increases in response to a rich diet after a period of DR (long-switch) across a panel of 11 DGRP lines (195 is reference).

	Full Model				Effect of DR			Long switch versus rich diet		
coefficient	estimate	exp	s.e.	p	estimate	exp	p	estimate	exp	p
DR	-1.723	0.179	0.157	**<0.001**						
long switch	0.973	2.645	0.160	**<0.001**						
105	-2.681	0.068	0.146	**<0.001**						
136	-1.969	0.140	0.152	**<0.001**						
217	-0.080	0.923	0.159	0.61						
239	0.899	2.457	0.156	**<0.001**						
335	0.190	1.209	0.155	0.22						
362	-1.022	0.360	0.155	**<0.001**						
441	-2.107	0.122	0.152	**<0.001**						
705	-1.861	0.156	0.154	**<0.001**						
707	1.073	2.925	0.156	**<0.001**						
853	-1.803	0.165	0.152	**<0.001**						
105 * DR	1.296	3.655	0.217	**<0.001**	-0.427	0.653	**0.049**			
136 * DR	1.995	7.355	0.213	**<0.001**	0.273	1.314	0.199			
217 * DR	0.622	1.863	0.219	**0.004**	-1.100	0.333	**<0.001**			
239 * DR	2.427	11.319	0.211	**<0.001**	0.704	2.022	**0.001**			
335 * DR	2.947	19.052	0.208	**<0.001**	1.225	3.403	**<0.001**			
362 * DR	0.343	1.409	0.219	0.12	-1.379	0.252	**<0.001**			
441 * DR	1.002	2.724	0.215	**<0.001**	-0.720	0.487	**0.001**			
705 * DR	0.602	1.826	0.217	**0.006**	-1.120	0.326	**<0.001**			
707 * DR	0.886	2.424	0.215	**<0.001**	-0.837	0.433	**<0.001**			
853 * DR	2.549	12.796	0.210	**<0.001**	0.827	2.286	**<0.001**			
105 * long switch	0.168	1.183	0.220	0.44				1.141	3.130	**<0.001**
136 * long switch	-0.381	0.683	0.222	0.086				0.592	1.807	**0.008**
217 * long switch	1.152	3.165	0.226	**<0.001**				2.125	8.370	**<0.001**
239 * long switch	0.870	2.387	0.224	**<0.001**				1.843	6.313	**<0.001**
335 * long switch	-0.305	0.737	0.221	0.17				0.668	1.950	**0.002**
362 * long switch	-0.982	0.375	0.223	**<0.001**				-0.009	0.991	0.968
441 * long switch	-0.172	0.842	0.220	0.43				0.800	2.226	**<0.001**
705 * long switch	-0.605	0.546	0.222	**0.006**				0.368	1.445	0.097
707 * long switch	0.188	1.206	0.218	0.39				1.160	3.191	**<0.001**
853 * long switch	0.117	1.124	0.225	0.6				1.090	2.973	**<0.001**

Table S8. Models run within each genotype testing for increases in response to a rich diet after a period of DR (long-switch).

	Estimates from individual models			
coefficient	estimate	exp	s.e.	p
105 DR	-0.501	0.149	0.606	**0.001**
136 DR	0.647	0.120	1.910	**<0.001**
195 DR	-1.737	0.197	0.176	**<0.001**
217 DR	-1.102	0.178	0.332	**<0.001**
239 DR	0.775	0.071	2.170	**<0.001**
335 DR	0.570	0.115	1.768	**<0.001**
362 DR	-1.201	0.130	0.301	**<0.001**
441 DR	-0.503	0.101	0.605	**<0.001**
705 DR	-0.935	0.095	0.393	**<0.001**
707 DR	-0.646	0.076	0.524	**<0.001**
853 DR	0.828	0.093	2.288	**<0.001**
105 long switch	1.258	0.156	3.520	**<0.001**
136 long switch	0.113	0.132	1.120	0.39
195 long switch	0.453	0.203	1.573	**0.025**
217 long switch	1.650	0.187	5.205	**<0.001**
239 long switch	1.483	0.103	4.406	**<0.001**
335 long switch	0.881	0.136	2.413	**<0.001**
362 long switch	0.145	0.137	1.157	0.29
441 long switch	0.313	0.109	1.368	**0.004**
705 long switch	0.329	0.102	1.389	**0.001**
707 long switch	0.854	0.083	2.348	**<0.001**
853 long switch	1.373	0.122	3.949	**<0.001**

Table S9. Effect of alternating DR and rich diets every 4 days (4-day switch) on longevity across 11 DGRP lines (195 is reference).

	Full Model				Effect compared to rich diet		
coefficient	estimate	exp	s.e.	p	estimate	exp	p
4-day switch	-0.140	0.869	0.114	0.22			
105	-2.476	0.084	0.096	**<0.001**			
136	-1.858	0.156	0.098	**<0.001**			
217	-0.065	0.937	0.100	0.52			
239	0.918	2.505	0.099	**<0.001**			
335	0.226	1.254	0.100	**0.024**			
362	-0.988	0.373	0.099	**<0.001**			
441	-1.969	0.140	0.097	**<0.001**			
705	-1.769	0.171	0.098	**<0.001**			
707	1.054	2.869	0.100	**<0.001**			
853	-1.697	0.183	0.098	**<0.001**			
105 * 4-day switch	0.644	1.904	0.159	**<0.001**	0.504	1.656	**0.002**
136 * 4-day switch	0.632	1.880	0.157	**<0.001**	0.492	1.635	**0.002**
217 * 4-day switch	0.490	1.632	0.158	**0.002**	0.350	1.419	**0.027**
239 * 4-day switch	1.053	2.866	0.157	**<0.001**	0.913	2.491	**<0.001**
335 * 4-day switch	1.026	2.789	0.158	**<0.001**	0.886	2.425	**<0.001**
362 * 4-day switch	-0.343	0.710	0.157	**0.029**	-0.483	0.617	**0.002**
441 * 4-day switch	0.163	1.177	0.158	0.3	0.023	1.023	0.885
705 * 4-day switch	-0.032	0.968	0.157	0.84	-0.172	0.842	0.273
707 * 4-day switch	0.153	1.165	0.158	0.33	0.013	1.013	0.935
853 * 4-day switch	0.365	1.441	0.157	**0.02**	0.225	1.253	0.152

Table S10. Effect of alternating DR and rich diets every 4 days (4 day switch) on mortality at each diet, across 11 DGRP lines (195 is reference).

								Compared to continious diets					
	Full Model				Effect of DR			4 day switch at rich diet			4 day switch at DR		
coefficient	estimate	exp	s.e.	p	estimate	exp	p	estimate	exp	p	estimate	exp	p
DR	-1.378	0.252	0.129	**<0.001**									
4-day switch	0.305	1.357	0.132	**0.02**									
4-day switch * DR	-0.078	0.925	0.164	0.63									
105	-2.649	0.071	0.109	**<0.001**									
136	-1.907	0.148	0.111	**<0.001**									
217	-0.064	0.938	0.115	0.58									
239	0.907	2.476	0.113	**<0.001**									
335	0.227	1.255	0.114	**0.046**									
362	-1.024	0.359	0.113	**<0.001**									
441	-2.046	0.129	0.111	**<0.001**									
705	-1.846	0.158	0.112	**<0.001**									
707	1.051	2.859	0.113	**<0.001**									
853	-1.733	0.177	0.111	**<0.001**									
105 * DR	1.171	3.224	0.180	**<0.001**	-0.207	0.813	0.248						
136 * DR	1.804	6.071	0.178	**<0.001**	0.426	1.531	**0.017**						
217 * DR	0.822	2.275	0.178	**<0.001**	-0.556	0.574	**0.002**						
239 * DR	2.046	7.740	0.179	**<0.001**	0.668	1.951	**<0.001**						
335 * DR	2.104	8.197	0.178	**<0.001**	0.726	2.066	**<0.001**						
362 * DR	0.307	1.359	0.179	0.087	-1.071	0.343	**<0.001**						
441 * DR	0.994	2.701	0.179	**<0.001**	-0.384	0.681	**0.031**						
705 * DR	0.527	1.695	0.178	**0.003**	-0.851	0.427	**<0.001**						
707 * DR	0.797	2.218	0.179	**<0.001**	-0.581	0.559	**0.001**						
853 * DR	2.090	8.086	0.178	**<0.001**	0.712	2.038	**<0.001**						
105 * 4-day switch	0.530	1.699	0.184	**0.004**				0.835	2.305	**<0.001**			
136 * 4-day switch	0.648	1.912	0.182	**<0.001**				0.953	2.595	**<0.001**			
217 * 4-day switch	0.372	1.451	0.183	**0.042**				0.677	1.969	**<0.001**			
239 * 4-day switch	1.019	2.771	0.182	**<0.001**				1.324	3.759	**<0.001**			
335 * 4-day switch	0.451	1.569	0.186	**0.015**				0.756	2.129	**<0.001**			
362 * 4-day switch	-0.285	0.752	0.182	0.12				0.020	1.020	0.914			
441 * 4-day switch	-0.158	0.854	0.184	0.39				0.148	1.159	0.421			
705 * 4-day switch	-0.185	0.831	0.182	0.31				0.120	1.127	0.511			
707 * 4-day switch	0.167	1.182	0.183	0.36				0.472	1.603	**0.01**			
853 * 4-day switch	-0.140	0.869	0.185	0.45				0.165	1.179	0.372			
105 * 4-day switch * DR	-0.580	0.560	0.223	**0.009**							0.177	1.194	0.427
136 * 4-day switch * DR	-1.735	0.176	0.226	**<0.001**							-0.859	0.423	**<0.001**
217 * 4-day switch * DR	-0.272	0.762	0.223	0.22							0.327	1.387	0.142
239 * 4-day switch * DR	-1.846	0.158	0.225	**<0.001**							-0.600	0.549	**0.008**
335 * 4-day switch * DR	-0.400	0.670	0.219	0.068							0.277	1.319	0.207
362 * 4-day switch * DR	-0.838	0.433	0.238	**<0.001**							-0.896	0.408	**<0.001**
441 * 4-day switch * DR	0.179	1.196	0.219	0.41							0.249	1.282	0.256
705 * 4-day switch * DR	-0.050	0.951	0.222	0.82							-0.009	0.992	0.969
707 * 4-day switch * DR	-0.910	0.403	0.232	**<0.001**							-0.516	0.597	**0.026**
853 * 4-day switch * DR	-0.477	0.621	0.218	**0.028**							-0.390	0.677	0.073

Table S11. Interval models run within each genotype testing for differential effects of diet in the 4-day switch dietary regime.

	Estimates from individual models			
coefficient	estimate	exp	s.e.	p
105 DR	-0.162	0.129	0.850	0.21
136 DR	0.576	0.134	1.780	**<0.001**
195 DR	-1.464	0.197	0.231	**<0.001**
217 DR	-0.695	0.132	0.499	**<0.001**
239 DR	0.697	0.075	2.008	**<0.001**
335 DR	0.516	0.128	1.675	**<0.001**
362 DR	-0.954	0.105	0.385	**<0.001**
441 DR	-0.466	0.140	0.628	**0.001**
705 DR	-0.741	0.078	0.476	**<0.001**
707 DR	-0.483	0.053	0.617	**<0.001**
853 DR	0.738	0.106	2.093	**<0.001**
105 4-day switch	0.789	0.132	2.201	**<0.001**
136 4-day switch	1.090	0.135	2.974	**<0.001**
195 4-day switch	0.265	0.195	1.303	0.18
217 4-day switch	0.756	0.134	2.131	**<0.001**
239 4-day switch	1.367	0.078	3.925	**<0.001**
335 4-day switch	0.351	0.137	1.421	**0.01**
362 4-day switch	0.065	0.106	1.068	0.54
441 4-day switch	0.139	0.143	1.149	0.33
705 4-day switch	0.171	0.081	1.186	**0.036**
707 4-day switch	0.543	0.054	1.720	**<0.001**
853 4-day switch	0.176	0.113	1.192	0.12
105 4-day switch * DR	-0.686	0.155	0.503	**<0.001**
136 4-day switch * DR	-1.965	0.165	0.140	**<0.001**
195 4-day switch * DR	0.018	0.221	1.018	0.94
217 4-day switch * DR	-0.166	0.158	0.847	0.29
239 4-day switch * DR	-1.956	0.119	0.141	**<0.001**
335 4-day switch * DR	-0.292	0.150	0.746	0.052
362 4-day switch * DR	-1.023	0.160	0.360	**<0.001**
441 4-day switch * DR	0.199	0.159	1.220	0.21
705 4-day switch * DR	-0.242	0.116	0.785	**0.037**
707 4-day switch * DR	-1.113	0.120	0.329	**<0.001**
853 4-day switch * DR	-0.579	0.129	0.560	**<0.001**

Table S12. Models run within each genotype testing for increases in response to a rich diet after a period of DR (long-switch) but within lines that showed starvation only, and with DR as reference category.

	Estimates from individual models			
coefficient	estimate	exp	s.e.	p
136 rich diet	-0.647	0.524	0.120	**<0.001**
239 rich diet	-0.775	0.461	0.071	**<0.001**
335 rich diet	-0.570	0.566	0.115	**<0.001**
853 rich diet	-0.828	0.437	0.093	**<0.001**
136 long switch	0.113	1.120	0.132	0.39
239 long switch	1.483	4.406	0.103	**<0.001**
335 long switch	0.881	2.413	0.136	**<0.001**
853 long switch	1.373	3.949	0.122	**<0.001**

Table S13. Interval models run within each genotype testing for differential effects of diet in the 4-day switch dietary regime, but within lines that showed starvation only, and with DR as reference category.

	Estimates from individual models			
coefficient	estimate	exp	s.e.	p
136 rich diet	-0.576	0.562	0.134	**<0.001**
239 rich diet	-0.697	0.498	0.075	**<0.001**
335 rich diet	-0.516	0.597	0.128	**<0.001**
853 rich diet	-0.738	0.478	0.106	**<0.001**
136 4-day switch	-0.875	0.417	0.169	**<0.001**
239 4-day switch	-0.589	0.555	0.111	**<0.001**
335 4-day switch	0.059	1.061	0.148	0.69
853 4-day switch	-0.403	0.668	0.124	**0.001**
136 4-day switch * rich diet	1.965	7.135	0.165	**<0.001**
239 4-day switch * rich diet	1.956	7.071	0.119	**<0.001**
335 4-day switch * rich diet	0.292	1.340	0.150	0.052
853 4-day switch * rich diet	0.579	1.785	0.129	**<0.001**

Table S14. Linear model of estimates of (log-transformed) fecundity (from Quantifly) in the long-switch dietary treatment. A return to rich conditions from DR, resulted in reduced fecundity, rather than the predicted increase.

	Full model		
coefficient	estimate	s.e.	p
rich diet	2.281	0.038	**<0.001**
long switch	-0.367	0.037	**<0.001**
441	0.149	0.036	**<0.001**
853	0.010	0.035	0.768
age 45	0.040	0.041	0.339
age 46	0.028	0.042	0.512
age 47	-0.018	0.054	0.736
441 * long switch	-0.195	0.050	**<0.001**
853 * long switch	-0.043	0.050	0.4

Table S15. Linear model of estimates of (log-transformed) fecundity (from Quantifly), corrected for number of flies in the cage, in the long-switch dietary treatment. A return to rich conditions from DR, resulted in reduced fecundity, rather than the predicted increase. Note, this correction uses the census after egg-laying and thus overcorrects for mortality. Estimates compared are thus biased upwards, and provide the most sensitive test for an upregulation in response to dietary treatment.

	Full model		
coefficient	estimate	s.e.	p
Intercept	0.374	0.048	**<0.001**
long switch	-0.515	0.046	**<0.001**
441	0.079	0.046	0.092
853	0.041	0.044	0.355
age 45	0.049	0.052	0.349
age 46	0.060	0.053	0.262
age 47	0.132	0.067	0.058
441 * long switch	-0.158	0.063	**0.017**
853 * long switch	0.471	0.063	**<0.001**

Table S16. Mixed model (correcting for Cage) of estimates of (log-transformed) fecundity (from Quantifly) in the 4-day switching paradigm. Repeated short-term exposure to DR did not increase, but rather decreased fecundity, relative to a continuous rich diet.

	Full model		
coefficient	estimate	s.e.	p
Intercept	2.924	0.057	**<0.001**
DR	-0.642	0.030	**<0.001**
4-day switch	-0.131	0.047	**0.005**
136	0.028	0.032	0.39
195	0.092	0.033	**0.006**
217	0.018	0.032	0.569
239	-0.162	0.032	**<0.001**
335	-0.302	0.032	**<0.001**
362	-0.092	0.033	**0.005**
705	-0.570	0.032	**<0.001**
707	-0.021	0.032	0.513
853	-0.248	0.032	**<0.001**
Age 9	-0.046	0.059	0.444
Age 10	-0.073	0.054	0.171
Age 11	-0.040	0.055	0.474
Age 12	0.011	0.053	0.841
Age 13	-0.086	0.054	0.11
Age 14	-0.104	0.053	**0.049**
Age 15	-0.115	0.054	**0.032**
Age 16	-0.139	0.053	**0.009**
Age 17	-0.070	0.054	0.196
Age 18	-0.049	0.055	0.375
Age 19	-0.202	0.056	**<0.001**
Age 20	-0.212	0.056	**<0.001**
Age 21	-0.367	0.072	**<0.001**
4-day switch * DR	0.287	0.065	**<0.001**
136 * DR	-0.036	0.043	0.401
195 * DR	-0.114	0.044	**0.01**
217 * DR	-0.109	0.043	**0.011**
239 * DR	0.017	0.043	0.691
335 * DR	0.164	0.043	**<0.001**
362 * DR	0.072	0.043	0.09
705 * DR	0.422	0.043	**<0.001**
707 * DR	0.032	0.043	0.447
853 * DR	0.211	0.043	**<0.001**
136 * 4-day switch	-0.014	0.066	0.829
195 * 4-day switch	0.071	0.067	0.285
217 * 4-day switch	0.053	0.066	0.424
239 * 4-day switch	-0.029	0.066	0.656
335 * 4-day switch	0.015	0.066	0.818
362 * 4-day switch	-0.060	0.066	0.362
705 * 4-day switch	0.219	0.066	**0.001**
707 * 4-day switch	0.002	0.066	0.976
853 * 4-day switch	0.017	0.066	0.795
136 * 4-day switch * DR	0.215	0.092	**0.02**
195 * 4-day switch * DR	0.162	0.093	0.081
217 * 4-day switch * DR	0.166	0.092	0.071
239 * 4-day switch * DR	-0.048	0.092	0.601
335 * 4-day switch * DR	-0.023	0.092	0.805
362 * 4-day switch * DR	0.218	0.092	**0.018**
705 * 4-day switch * DR	-0.422	0.092	**<0.001**
707 * 4-day switch * DR	0.149	0.092	0.106
853 * 4-day switch * DR	0.112	0.092	0.225

Table S17. Mixed model (correcting for Cage) of estimates of (log-transformed) fecundity (from Quantifly), corrected for number of flies in the cage, in the 4-day switching paradigm. Repeated short-term exposure to DR did not increase, but rather decreased fecundity, relative to a continuous rich diet. Note, this correction uses the census after egg-laying and thus overcorrects for mortality. Estimates compared are thus biased upwards, and provide the most sensitive test for an upregulation in response to dietary treatment.

	Full model		
coefficient	estimate	s.e.	p
Intercept	0.812	0.056	**<0.001**
DR	-0.640	0.030	**<0.001**
4-day switch	-0.126	0.046	**0.006**
136	0.051	0.032	0.111
195	0.094	0.033	**0.004**
217	0.029	0.031	0.363
239	-0.124	0.031	**<0.001**
335	-0.180	0.032	**<0.001**
362	-0.057	0.032	0.08
705	-0.556	0.031	**<0.001**
707	0.048	0.032	0.133
853	-0.225	0.032	**<0.001**
Age 9	-0.053	0.059	0.37
Age 10	-0.089	0.053	0.094
Age 11	-0.059	0.055	0.283
Age 12	0.008	0.053	0.883
Age 13	-0.083	0.053	0.12
Age 14	-0.099	0.052	0.06
Age 15	-0.107	0.053	**0.045**
Age 16	-0.120	0.053	**0.022**
Age 17	-0.043	0.054	0.419
Age 18	-0.027	0.055	0.626
Age 19	-0.161	0.055	**0.003**
Age 20	-0.167	0.055	**0.003**
Age 21	-0.151	0.071	**0.034**
4-day switch * DR	0.297	0.064	**<0.001**
136 * DR	-0.027	0.042	0.517
195 * DR	-0.119	0.044	**0.007**
217 * DR	-0.114	0.042	**0.007**
239 * DR	0.074	0.042	0.081
335 * DR	0.170	0.042	**<0.001**
362 * DR	0.064	0.042	0.129
705 * DR	0.418	0.042	**<0.001**
707 * DR	0.000	0.042	0.992
853 * DR	0.213	0.042	**<0.001**
136 * 4-day switch	0.003	0.065	0.969
195 * 4-day switch	0.078	0.066	0.236
217 * 4-day switch	0.050	0.065	0.446
239 * 4-day switch	0.076	0.065	0.243
335 * 4-day switch	0.019	0.065	0.767
362 * 4-day switch	-0.062	0.065	0.342
705 * 4-day switch	0.217	0.065	**0.001**
707 * 4-day switch	-0.002	0.065	0.97
853 * 4-day switch	0.013	0.065	0.841
136 * 4-day switch * DR	0.178	0.091	0.051
195 * 4-day switch * DR	0.156	0.092	0.089
217 * 4-day switch * DR	0.164	0.091	0.072
239 * 4-day switch * DR	-0.060	0.091	0.511
335 * 4-day switch * DR	-0.030	0.091	0.738
362 * 4-day switch * DR	0.215	0.091	**0.018**
705 * 4-day switch * DR	-0.431	0.091	**<0.001**
707 * 4-day switch * DR	0.207	0.091	**0.023**
853 * 4-day switch * DR	0.098	0.091	0.283

Table S18. Effect of returning to a rich diet after a period of DR (long-switch) after ablation of the microbiome (Antibiotics on rich diet is reference).

	Full Model			
coefficient	estimate	exp (estimate)	s.e.	p
DR on Antibiotics	-0.894	0.409	0.181	**<0.001**
long switch on Antibiotics	1.313	3.717	0.142	**<0.001**
DR	-1.201	0.301	0.223	**<0.001**
Rich diet	-0.087	0.917	0.159	0.59

Table S19. Effect of returning to a rich diet after a period of DR (long-switch) with supplementation of water.

	Full Model			
coefficient	estimate	exp (estimate)	s.e.	p
DR with water supplementation	0.648	1.911	0.138	**<0.001**
long switch with water supplementation	0.385	1.470	0.126	**0.002**

Table S20. Effect of returning to a rich diet after a period of DR (long-switch) with flies in isolation in vials. Test from chi-square tests on proportions by age.

	Dead				Mortality rate				P value from proportion test	
Age	DR	Rich to DR	Rich	DR to Rich (long switch)	DR	Rich to DR	Rich	DR to Rich (long switch)	Rich versus DR	Rich versus long switch
28	0	0	5	16	0.000	0	0.100	0.320	0.063	**0.014**
30	0	0	8	5	0.000	0	0.178	0.152	**0.006**	1
32	1	0	16	13	0.020	0	0.432	0.464	**<0.001**	0.997
34	1	0	8	4	0.020	0	0.381	0.267	**<0.001**	0.72
36	1	0	8	2	0.020	0	0.667	0.200	**<0.001**	0.079
38	4	0	2	2	0.087	0	0.500	0.250	0.102	0.829
40	3	0	0	3	0.071	0	0.000	0.500	1	1
42	2	0	0	3	0.051	0	0.000	1.000	1	0.505

Table S21. Effect of switching from DR to rich food every 4 days (4-day switch) in males.

	Full Model			
coefficient	estimate	exp (estimate)	s.e.	p
DR	0.362	1.437	0.117	**0.002**
4-day switch	0.410	1.507	0.120	**0.001**
4-day switch * DR	-0.768	0.464	0.129	**<0.001**
